# A Systematic Review Comparing Experimental Design of Animal and Human Methotrexate Efficacy Studies for Rheumatoid Arthritis: Lessons for the Translational Value of Animal Studies

**DOI:** 10.3390/ani10061047

**Published:** 2020-06-17

**Authors:** Cathalijn Leenaars, Frans Stafleu, David de Jong, Maikel van Berlo, Tijmen Geurts, Tineke Coenen-de Roo, Jan-Bas Prins, Rosalie Kempkes, Janneke Elzinga, André Bleich, Rob de Vries, Franck Meijboom, Merel Ritskes-Hoitinga

**Affiliations:** 1 SYRCLE, Department for Health Evidence (Section HTA), Radboud Institute for Health Sciences, Radboud University Medical Centre, 6525 GA Nijmegen, The Netherlands; dhdejong1993@gmail.com (D.d.J.); maikelvberlo@gmail.com (M.v.B.); tijmengeurts@hotmail.com (T.G.); rosalie.kempkes@live.nl (R.K.); janneke.elzinga@wur.nl (J.E.); rob.devries@radboudumc.nl (R.d.V.); Merel.Ritskes-Hoitinga@radboudumc.nl (M.R.-H.); 2 Department of Population Health Science, Unit Animals in Science and Society, Utrecht University, 3508 TD Utrecht, The Netherlands; F.L.B.Meijboom@uu.nl; 3 Institute for Laboratory Animal Science, Hannover Medical School, 30625 Hannover, Germany; bleich.andre@mh-hannover.de; 4 Ethics Institute, Utrecht University, 3508 TC Utrecht, The Netherlands; F.R.Stafleu@uu.nl; 5 Central Animal Facility, Leiden University Medical Centre, 2300 RC Leiden, The Netherlands; t.coenen@coenenconsultancy.nl; 6 Biological Research Facility, The Francis Crick Institute, London NW1 1AT, UK; jan-bas.prins@crick.ac.uk

**Keywords:** Systematic review, experimental design, animal-to-human translation, rheumatoid arthritis, methotrexate

## Abstract

**Simple Summary:**

If we want to use animal studies to predict what will happen if we give a drug to humans, it makes sense to perform the animal studies as similarly to human studies as possible. For example, if animal tests of a drug only look at the effect of injecting the drug in young healthy animals, we cannot expect the results to be similar in human tests giving tablets to older patients who may have other diseases besides the one for which they receive the drug. We did an in-depth analysis of how 147 animal and 512 human studies of the drug methotrexate for rheumatoid arthritis were performed. Important differences were present, for example, animal studies used more males, while rheumatoid arthritis occurs more in females. We calculated the human-equivalent age of the animals, and they were on average younger than humans. Many studies did not fully report the way the experiments were performed. In spite of these differences, the drug methotrexate works well against rheumatoid arthritis in animal models and humans. Further (literature) research is still needed; we do not yet understand when we can reliably predict human effects from animal studies.

**Abstract:**

Increased awareness and understanding of current practices in translational research is required for informed decision making in drug development. This paper describes a systematic review of methotrexate for rheumatoid arthritis, comparing trial design between 147 animal and 512 human studies. Animal studies generally included fewer subjects than human studies, and less frequently reported randomisation and blinding. In relation to life span, study duration was comparable for animals and humans, but included animals were younger than included humans. Animal studies often comprised males only (61%), human studies always included females (98% included both sexes). Power calculations were poorly reported in both samples. Analyses of human studies more frequently comprised Chi-square tests, those of animal studies more frequently reported analyses of variance. Administration route was more variable, and more frequently reported in animal than human studies. Erythrocyte sedimentation rate and c-reactive protein were analysed more frequently in human than in animal studies. To conclude, experimental designs for animal and human studies are not optimally aligned. However, methotrexate is effective in treating rheumatoid arthritis in animal models and humans. Further evaluation of the available evidence in other research fields is needed to increase the understanding of translational success before we can optimise translational strategies.

## 1. Introduction

In these times of focus on animal-free alternatives for drug development, we need to ensure progress in fields of unmet patient needs; when it is possible to replace animal experiments by alternative tests without animals, we should of course do so, but research may still be necessary before alternatives become available. For informed decision making, we require increased awareness and understanding of current practices in translational research. Our recent systematic scoping review shows that animal-to-human translational success rates vary from 0% to 100% and that they did not clearly correlate with species, study size, methods used to calculate translational success, overall research field, or year of publication [1]. In our work, we focus on direct animal to human translation, which we believe to be restricted to animal and human experiments preceding market introduction. Translation from clinical trials to clinical practice is another phase of the drug-developmental process where many compounds fail but are considered out of scope for this special issue.

One of the factors possibly contributing to translational success and failure that we could not test in our preceding scoping review is experimental design. For example, if a drug is preclinically only tested with parenteral administration to young animals without comorbidities, extrapolation to oral administration in older patients with comorbidities is not warranted. Besides, implementation of randomisation and blinding to limit confounding of the results by different types of bias, (e.g., expectations of participants and investigators), and selecting appropriate analyses and sample size to answer the primary research question are necessary to guarantee internal validity of the research results before external validity can be assessed. If a clear relationship exists between study quality and translational success, we should drastically and urgently improve animal and human study quality. If translational success cannot be predicted at all, we should redirect the focus to alternative approaches based on human biology.

Systematic reviews (SRs) of animal studies consistently show an unclear or high risk of bias for the majority of the included studies (e.g., [2]). Besides, animal studies are often poorly aligned with clinical trials (e.g., [3]). A recent SR of 123 clinical and experimental studies also showed that poor experimental design (e.g., not randomising, not blinding and insufficient power) is common, and that inflated effect sizes were more frequently observed in both equine and human trials with a high risk of bias [4]. Others have highlighted specific problems with sample size [5] and randomisation and blinding [6] in animal studies.

As described before, the level of concordance between clinical and preclinical trial design is relevant for future drug development projects, but a clear overview of the experimental designs used in clinical trials and animal studies and the differences between them is lacking. Therefore, we decided to perform an SR comparing trial designs between animal and human studies. Because the total body of evidence is too large to analyse in this manner, we started with a case study. We selected rheumatoid arthritis (RA) for this case study because it is a well-defined research field with adequate numbers of animal and human studies to analyse. Besides, there is an urgency to optimise practices in this field, as RA animal models cause substantial discomfort to the experimental animals, while further drug development is still necessary to address unmet patient needs. Moreover, the available animal models have their limitations, and several animal-free technologies, comprising patient-derived cells, 3D cell culture systems, computational models, omics and large-scale epidemiological studies, can be used and are developed further [7].

To create a manageable homogeneous sample, we restricted our SR to methotrexate (MTX); a widely-used classical disease-modifying anti-rheumatic drug (DMARD). As this drug is generally effective [8], it is also often administered in clinical and preclinical trials as a positive control.

## 2. Methods

The protocol for this SR was posted on the SYRCLE website on the 19th of February 2016 [9], before we started screening of the papers retrieved from the searches for inclusion.

### 2.1. Paper Search and Selection

The search and selection are fully described in the original protocol [9]. In brief, we searched PubMed and EmBase for animal and human studies of MTX in RA on the 19th of February 2016. Searches comprised title-, abstract- and keywords, and internal thesaurus terms (Medical Subject Headings or Emtree terms). We combined the three search components (animals including humans, MTX and RA) with the Boolean operator “AND”. The full search strategy for each of the components is provided for both databases in Supplementary Materials Table S1.

Duplicates were manually removed in Endnote. Unique references were imported into the Early Review Organising Software (EROS; Institute of Clinical Effectiveness and Health Policy, Buenos Aires, Argentina) for screening in two separate phases: first, titles and abstracts, and second, full texts. Screening was performed by two (out of five) independent reviewers and discrepancies were resolved by discussion between them.

We included all studies in which MTX was administered to RA patients or RA animal models in our ongoing review. We excluded studies on other diseases than RA, studies that were not in vivo, studies that did not administer MTX (or only had treatment groups co-administering MTX with other experimental drugs), studies that did not analyse efficacy (e.g., safety studies), and publications that did not contain new data (e.g., reviews and editorials).

### 2.2. Data Extraction and Cleaning

Data extraction was performed according to our protocol and comprised bibliographic details, study design characteristics, animal model and human patient population characteristics, MTX schedule and dose, and all outcome measures for which results were reported. Data extraction and quality control were performed in Excel by seven of the authors. Distribution of the papers over the data extractors was based on random lists (generated on random.org). However, to allow for exploratory interim analyses, separate lists were used for animal and human studies, and some extractors preferentially extracted from animal or human studies. Two exploratory interim analyses were performed. The first compared 50 animal and 50 human papers (data extracted by DdJ). The second analysed the animal studies in more detail (additional animal data extracted by TG). The relative contribution of the different extractors to the overall dataset is shown in the results section (Section 3.2 on bibliographic details, including a bar plot). As species cannot be blinded during data extraction, we did not expect this distribution of the work to further affect the confounding of the outcomes.

Study locations (locations where the study was reportedly performed, educated guesses were only made if all authors had affiliations in the same country) were analysed at the country level using country codes (ISO3) developed by the International Organization for Standardization. Locations that were only specified at higher levels (e.g., Europe or Latin America) were not included in location analyses.

### 2.3. Data Analysis

To maximise our sample size, studies in languages other than English were included as far as possible in all analyses. We translated the relevant details from Dutch, French, German, Italian and Spanish to English, and extracted information from English abstracts, figures, tables and captions for publications in Chinese, Japanese, Russian, Polish, Portuguese and Serbian. Where percentages of reported and not reported details do not add up to 100%, the difference is explained by our uncertainty on the reporting of these details in languages that we do not understand well enough.

For ease of analysis, and to prevent larger papers with multiple comparable experiments skewing the results, the unit of analysis was the publication, if a paper reported more than one experimental group, we used median values for the analyses. If ranges were reported, we included the median values in our analyses. For comparisons of animal and human age of the included population, animal ages were very crudely converted to human years. Lacking proper scientific evidence for these conversions, we based ourselves on popular resources for estimations (http://www.age-converter.com/). For mice, 1 month was considered equivalent to 14 human years; for rats, 1 month was considered equivalent to 8 human years, for rabbits, 6 months was considered equivalent to 16 human years. If only bodyweight was provided in the included paper, age was estimated based on growth curves from the Charles River and Jackson laboratories websites.

Analyses were performed in R, version 3.6.3—“Holding the Windsock” [10] via RStudio, version 1.2.5033. Data were imported using the readxl package [11], organised and analysed using the dplyr [12] and tm [13] packages, and visualised using the ggplot2 [14], wordcloud [15] and rworldmap [16] packages. With one exception, no statistical hypothesis-testing was performed, mainly because the data are observational, and this study is explorative. Besides, the number of variables is so large that false-positives would be expected. The exception is publication date, for which some authors observed a difference between animal and human studies in the boxplot, while others did not. To be sure, a two-sample Wilcoxon’s rank-sum test was performed in R using the wilcox.test function (paired = FALSE).

Based on the authors’ experience, we were expecting reporting of experimental details to be better in human than in animal studies, and designs to be more variable in animal than in human studies. Other than that, we had no a priori expectations.

### 2.4. Protocol Deviations

In our protocol, we describe two research questions: first, “are the experimental designs of the pre-clinical animal studies comparable to those of the clinical trials?” and second, “are the improvements (i.e., reductions in swelling, pain and bone and cartilage damage) found in RA animal models comparable with the improvements found in patients?”. Several publications have already described meta-analyses directly comparing human and animal outcomes for other diseases and interventions [17,18,19,20]. As the number of papers included in our SR was larger than anticipated, we restricted ourselves to answering the first review question, which, as far as we know, has not been answered before. As we were thus not performing the considered meta-analysis on the effects of MTX, we omitted our risk of bias assessment. We did analyse the levels of reporting of e.g., randomization and blinding. Because of restricted resources, we also did not retrieve additional references from reference lists of included studies and relevant reviews.

The following data were extracted from the included papers, but not analysed for this publication: animal handling, number of animals per cage, laboratory temperature and humidity, lighting regime, habituation period after arrival, species, strain, substrain, line, type of RA model and details of model induction. These animal study characteristics will be described in a future publication comparing the different RA animal models, for which a separate SR protocol was written, which was posted on the SYRF website on the 24th of August 2018 [21]. General procedures for RA animal models have recently been published [22].

## 3. Results

### 3.1. Paper Flow

**Figure 1:**
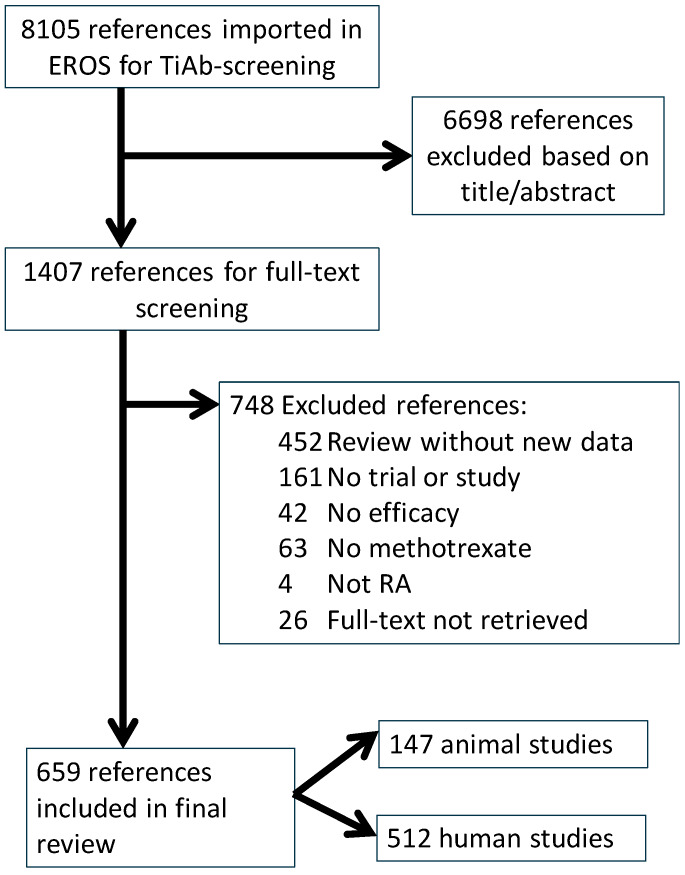
Paper flow.

Our search in Pubmed retrieved 3238 references, the search in Embase retrieved 6305. After duplicate removal, 8105 unique references remained. After screening, 659 references were included in this review, 147 on animals and 512 on humans. The flow of papers through the different phases and the reasons for exclusion in the full-text screening phase are shown in Figure 1. A list of the included papers is provided in Supplementary Materials Document S2.

### 3.2. Bibliographic Details

**Figure 2:**
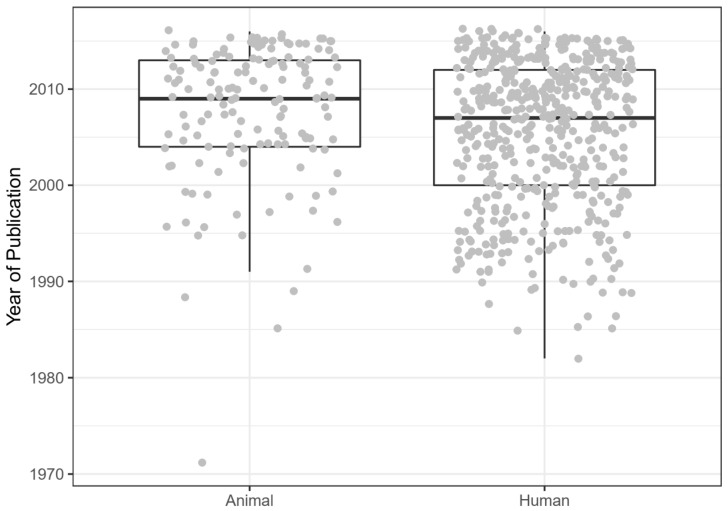
Publication dates for animal and human studies.

Overall, with 96 papers, the most popular journal in this data set is Arthritis and rheumatism, followed by the Annals of the Rheumatic Diseases (82 included papers) and the Journal of Rheumatology (52 included papers). The publication date ranged from 1971 to 2016 for animal studies, and from 1982 to 2016 for human studies. On average, animal studies were performed more recently than human studies (W = 44,556, *p* < 0.001; Figure 2).

**Figure 3:**
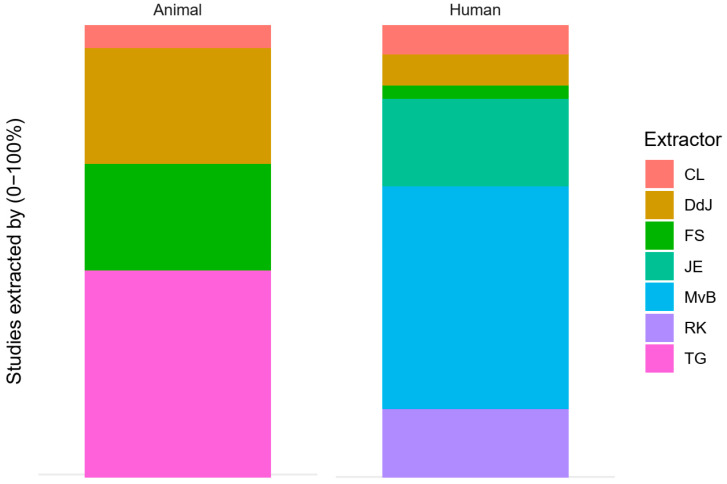
Data extractors. (**a**) Extractors for animal studies; (**b**) extractors for human studies.

Data were extracted by seven extractors. Their relative contribution to the extraction of animal and human studies is shown in Figure 3. TG extracted data exclusively from animal studies, while JE, RK and MvB extracted data exclusively from human studies. As explained in Section 2.2, we did not expect this to affect our results.

**Figure 4:**
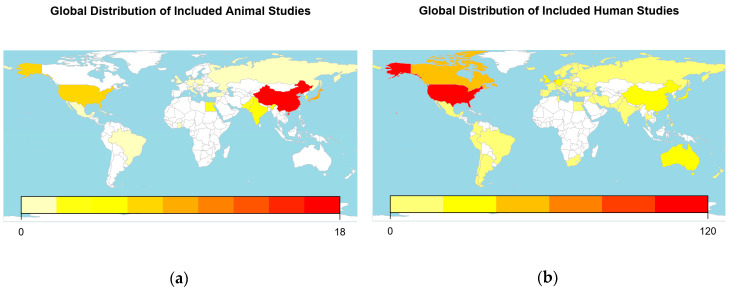
Geographic location. (**a**) World map depicting the locations where the included animal studies were performed; (**b**) world map depicting the locations where the included human studies were performed. The colour reflects the number of studies as indicated in the legend.

Most included papers were written in English. Besides, we included both animal and human studies in Chinese (k = 31), Japanese (k = 5) and Russian (k = 10), and human studies in Dutch (k = 1), French (k = 1), German (k = 6), Italian (k = 1), Polish (k = 3), Portuguese (k = 1), Serbian (k = 1) and Spanish (k = 1). Both animal and human studies were performed all over the world; the distribution is shown in Figure 4.

### 3.3. Experimental Design in Animal and Human Trials

#### 3.3.1. General Experimental Design

**Figure 5:**
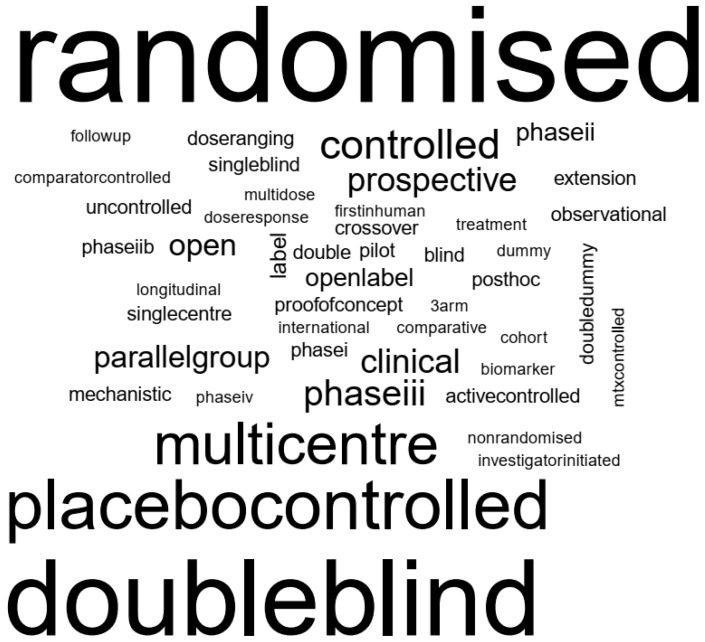
Impression of study design descriptions of the included human studies (word cloud). All text has been converted to lower-case and punctuation was removed (e.g., “Phase-III” now reads “phaseiii”). The most frequently used terms to describe human study design are: randomised (k = 329), double-blind (k = 248), placebo-controlled (k = 147) and multicentre (k = 113).

The included animal studies were all interventional experiments. Human studies showed more variation in overall study design. An impression of the words used to describe the experimental designs in human studies is shown as a word cloud in Figure 5.

**Figure 6:**
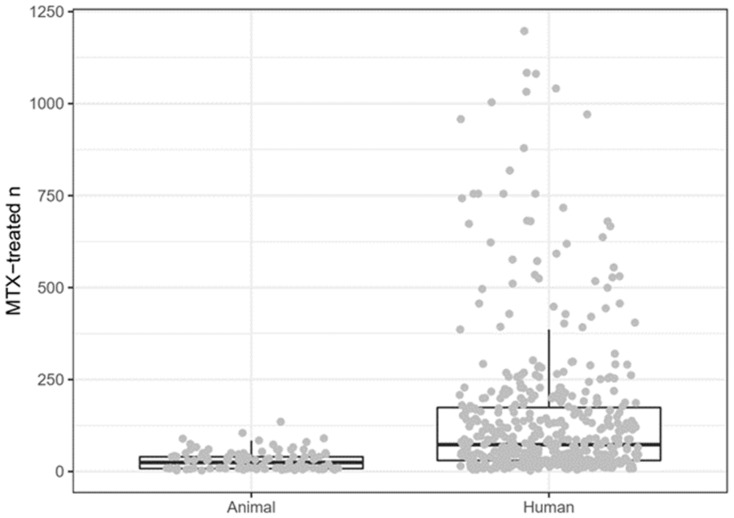
Number of methotrexate-treated subjects in animal and human studies. MTX: Methotrexate.

Overall numbers of subjects per study (n) ranged from 8 to 283 for animal studies (median: 40), and from 8 to 2550 for human studies (median: 144). The number of experimental subjects receiving exclusively MTX as the experimental treatment ranged from 3 to 135 per study (median: 24.5) for animal studies, and from 2 to 1197 (median: 73) for human studies (Figure 6). The number of centres in which the animal studies were performed was not specifically indicated in any of the included studies, but probably all animal studies were single-centre. Human studies were performed in 1–209 centres (median: 4), 101 human studies were single-centre.

Animal studies generally implemented a control group not receiving treatment (we were not sure of this for only 4 out of the 147 studies). In 200 human studies, a control group without active treatment was described, 255 human studies were either uncontrolled or used MTX as the active control for another drug. We included all studies where MTX was the single active treatment in at least one of the treatment groups, we did not restrict to studies specifically investigating MTX.

Animal studies lasted from 0.1 to 30 months (median: 1 month), human studies lasted from 0.1 to 132 months (median: 12 months). As a percentage of life span, the study duration for animal and human studies is considered fairly comparable. Animal studies reported 1–30 repeated measurements (median: 7) and human studies reported 1–63 (median: 5).

**Figure 7:**
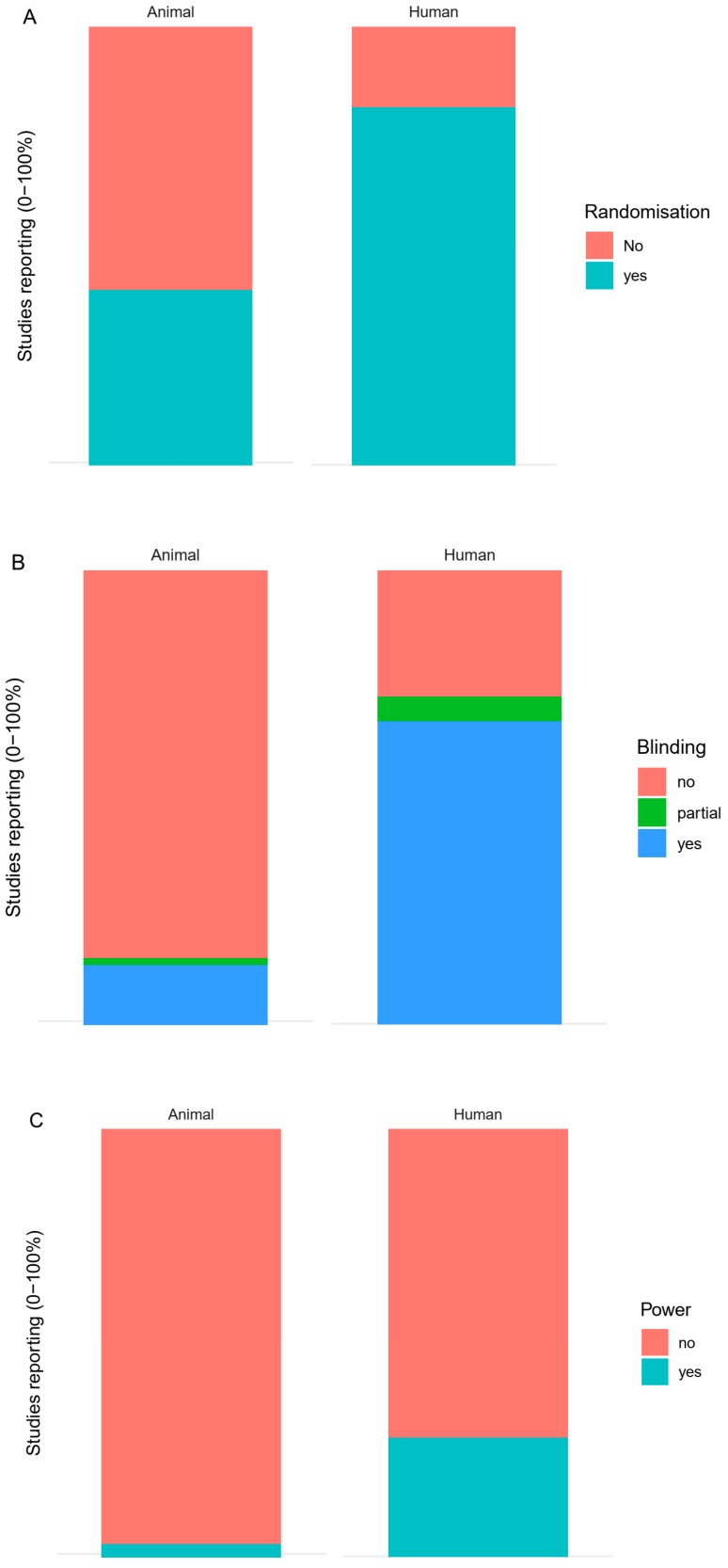
(**A**) Randomisation in animal and human studies; (**B**) blinding in animal and human studies; (**C**) power calculations in animal and human studies.

Randomisation (mainly of treatment allocation) was reported for 56 animal studies (38%) and for 401 human studies (78%, Figure 7A). Randomisation was not reported for 84 animal studies (57%) and 90 human studies (18%). At least partial blinding was reported for 19 animal studies (13%) and for 348 human studies (68%, Figure 7B). Blinding was not reported for 111 animal studies (76%) and 134 human studies (26%, Figure 7B). Power calculations were reported for 4 animal studies (3%) and for 131 human studies (26%, Figure 7C). Power calculations were not reported for 126 animal studies (86%) and for 334 human studies (65%). Refer to Section 2.3 for an explanation of the uncertainty causing percentages to not add up to 100% (we included studies in languages that we do not, or not fully, understand).

**Figure 8:**
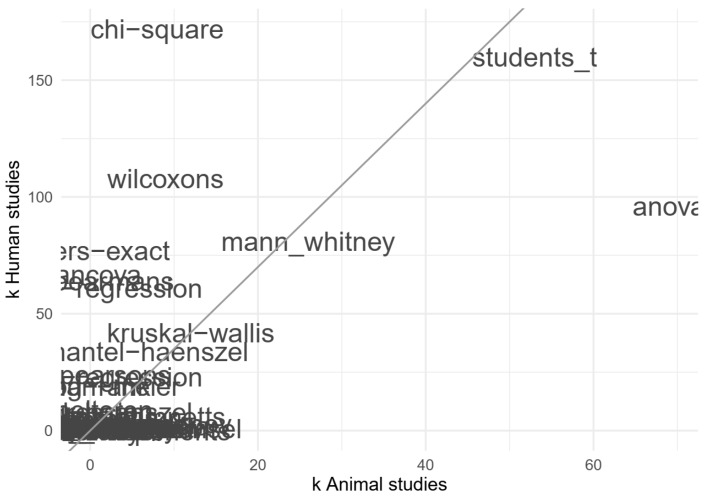
Impression of the reported frequencies of specific statistical analyses in animal and human studies; e.g., the ANOVA (analysis of variance test) was reportedly used in 69 animal studies (on the x-axis) and 96 human studies (on the y-axis). The illegible part in the lower-left corner reflects a large number of rare analyses that are not particularly relevant. The diagonal line reflects the ratio of included human vs. animal studies.

The most frequently reported analyses for animal studies were the analysis of variance (k = 69), the Student’s *t*-test (k = 53) and the Mann–Whitney test (k = 26, Figure 8). The most frequently reported analyses for human studies were the Chi-square test (k = 172), the Student’s *t*-test (k = 160) and Wilcoxon’s tests (either the signed-rank or the rank-sum test, k = 108, Figure 8). Note that most studies used multiple statistical tests, which were all included in these analyses.

#### 3.3.2. Tested Populations

**Figure 9:**
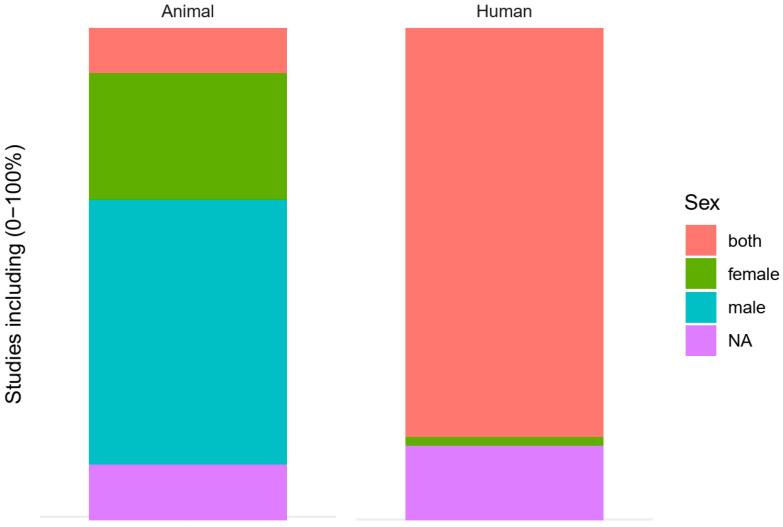
Sex in animal and human studies. NA: data not available.

512 studies were on human subjects, 147 on animals. Of the 147 animal studies, 99 were on rats, 43 on mice, 3 on both rats and mice and 2 on rabbits. Of the animal studies, 12 included both sexes, 34 were restricted to female animals and 71 to male animals (Figure 9). For 15 animal studies, we were not sure, and 15 other studies did not report the sex of the animals. Of the human studies, 411 included both sexes, 9 were restricted to females and none were restricted to males only (Figure 9). For 17 human studies, we were not sure, and 75 human studies did not report the sex of the participants.

Age ranged from 9 to 52.2 human-equivalent years for animal studies (median: 16 human-equivalent years) and from 10.2 to 76.5 years for human studies (median: 52, Figure 10).

**Figure 11:**
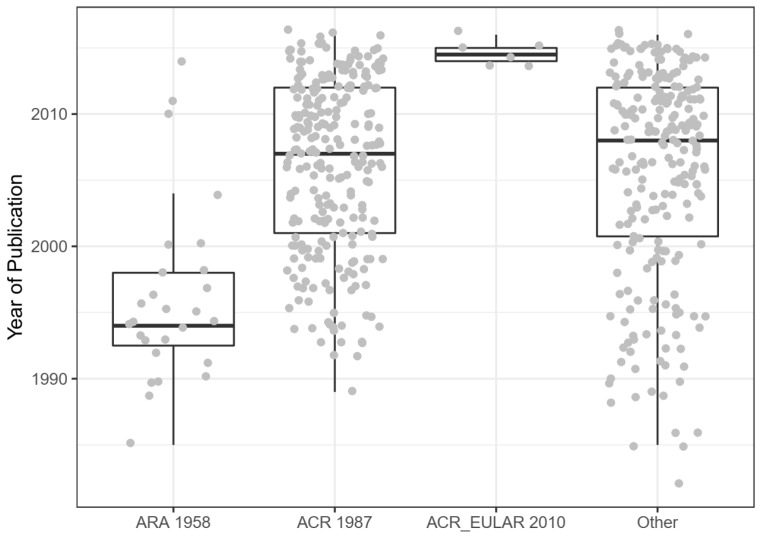
Publication date of human studies by diagnostic criteria. ACR 1987 criteria were also referred to as ARA 1987 criteria. Studies grouped under “other” reported the use of other criteria, or reported combinations of criteria, or did not report using specific criteria, or were in a language that the authors did not understand.

In animal studies, most (127; 86%) do not specifically indicate studying early or late disease. Many human studies are restricted to early (116) disease, some to late disease (9), and only 4 indicated the studying of both. Of the human studies, 344 specified restriction to active disease. Most human studies specified diagnostic criteria, and the criteria formulated by the American College of Rheumatology (previously the American Rheumatism Association) were commonly used. These clinical diagnostic criteria have been revised over time [23], which is reflected in the human studies in this sample; e.g., the six studies reporting the use of the most recently revised criteria were all published after 2013 (Figure 11). Of the 512 human studies, 156 reported excluding certain comorbidities, and 18 reported including specific comorbidities. None of the included animal studies modelled comorbid disease.

Most animal studies used treatment-naïve animals; one animal study described initial treatment besides MTX [24]. For human studies this was the reverse; only three included studies were restricted to treatment-naïve subjects and 222 studies specified various allowed preceding treatments. Two animal studies studied MTX-resistant animals [25,26], 114 human studies specified including partially or fully MTX-resistant subjects. Comedication was only administered in the one animal study described above but was explicitly allowed in 328 human studies (implicitly probably in more).

#### 3.3.3. MTX Interventions

MTX administration was an inclusion criterion for this review. The MTX dosing schedule was highly variable though. For animal studies, the most frequently reported dose schedules were daily (k = 36), weekly (k = 20) and twice a week (k = 17). In human studies, the most common dose schedule was weekly (k = 315), none of the other dose schedules were reported more than two times. In 10 animal studies and in one human study a single dose was given. Cumulative doses could not be calculated for the majority of the studies because of missing information and variable dosing schedules.

**Figure 12:**
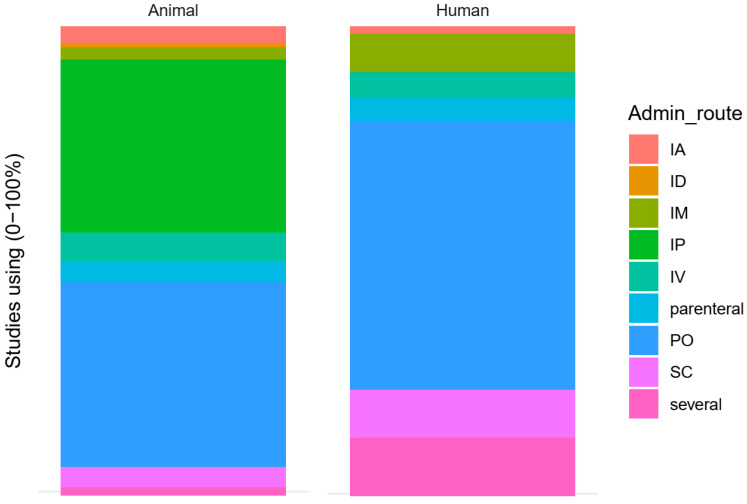
Route of administration for included animal and human studies. IA: Intra-articular; ID: intradermal; IM: intramuscular; IP: intraperitoneal; IV: intravenous; PO: per os; SC: subcutaneous.

Administration route was reported for 115 animal (78%) and 247 human (48%) studies. Administration route was not reported for 24 animal (16%) and 245 human (48%) studies. The different administration routes are shown by species in Figure 12. Note that when the authors specified “injection” or “infusion” without further information, we classified this as parenteral administration. In animals, administration per os comprises e.g., administration in drinking water [27], intragastric [28,29,30,31], gavage [32,33] and gastric tube [34,35].

MTX was compared with various other treatments. In animal studies, the most common comparator was non-treated controls (k = 116), followed by other drugs co-administered with MTX (k = 17), saline (k = 11) and vehicle (k = 6). In human studies, the most common comparator was other drugs co-administered with MTX (k = 285), followed by placebo (k = 53), etanercept (k = 47) and adalimumab (k = 42). For 126 human studies, the MTX group received a placebo besides the MTX, as a positive control condition for combined treatment, with MTX and another drug.

#### 3.3.4. Analysed Outcome Measures

**Figure 13:**
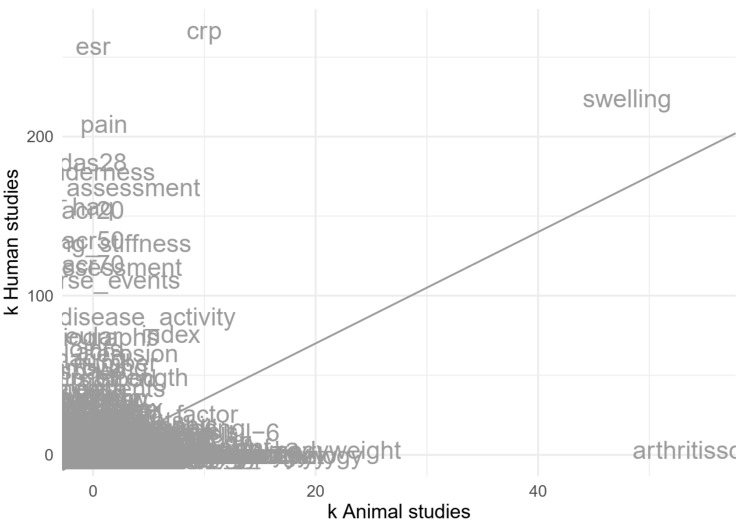
Impression of outcome measures in animal and human studies. For example, CRP (C-reactive protein) is reported in 10 animal (x-axis) and 267 human (y-axis) studies. The illegible part in the lower-left corner reflects a large number of rare outcomes that are not particularly relevant. The diagonal line reflects the ratio of included human vs. animal studies.

In animal studies, the outcome measures most frequently analysed were arthritis scores (k = 68), paw swelling (k = 43) and weight (k = 27). The most frequently reported biochemical outcome measures (pooled over specimen types) were TNF-alpha (k = 29) and IL-6 (k = 15). In human studies, the most frequently reported outcome measures were C-reactive protein (k = 267), the erythrocyte sedimentation rate (k = 257), swelling (k = 224), pain (k = 208) and the DAS28 (disease activity score, k = 184). Relative reporting of outcome measures in animal and human studies is shown in Figure 13.

## 4. Discussion

In this paper, we compared the experimental design of 147 animal and 512 human studies of MTX for the treatment of RA. The animal and human studies are roughly comparable for bibliographic details, although the relative amounts of studies performed in different countries may vary and on average, the animal studies have been published somewhat later than the human studies.

Concerning the global distribution of study locations, the number of observations of animal studies per country is low (too low for reliable statistics). The interpretation is further complicated by the commonness of multi-centre human studies, while none of the included animal studies were reportedly performed in multiple locations. This resulted in only two countries (China and Japan) hosting more than 10 of the included animal studies, while 16 countries hosted more than 10 of the included human studies. Furthermore, if study location was not specifically mentioned, but all the authors had an affiliation in the same country, we listed this country as an educated guess. Moreover, some of the studies may have been performed as contract research. If contracting was not described in the publication, this would confound our location data.

The difference in publication dates may be surprising, as one would expect the human studies to be based on the animal studies. The included publication records for MTX start in 1971 with only a single animal study [36] preceding the first human study in 1982 [37]. However, MTX has been available for clinical use since 1951 [37]. Market access thus preceded the first included scientific publication by 20 years. At least three factors contribute to this observation.

First, in the field of RA, many clinically useful drugs have been identified serendipitously, after market introduction for another disorder [38,39]. MTX is no exception; it was first clinically tested as a treatment for childhood leukaemia and used off-label to treat RA [38]. MTX only received FDA-approval for RA treatment in 1988 [40]. Animal studies are still performed after market introduction of a drug when they can be part of “reverse translational” strategies and help to elucidate pathological mechanisms. In this case, the introduction of MTX for RA was based on clinical observations, no RA-specific animal trials were performed beforehand.

Second, our searches and inclusion criteria focussed on RA and MTX, and experiments for other disorders were excluded. Our search strings for MTX did not include title-abstract-keyword searching for the older synonym “amethopterin”, the historical name for this compound [38]. This term is included in the thesaurus (MeSH/Emtree) indexing for PubMed and Embase; we did retrieve the indexed papers, but our sample is probably skewed towards newer publications for those that were not indexed. Future searches for MTX should add this term.

Third, the small difference in publication date may reflect the increased focus on publications in evaluating scientific productiveness, which results in drastically increasing numbers of scientific papers being published each year. Older animal studies may have been published less frequently than older human studies. While there currently may be too much emphasis on publications to evaluate scientists’ performance, the lack of early data in the public domain prevents their reuse in ongoing work. Besides efforts to increase public availability of research data from ongoing studies (with trial registries), meta-research would benefit from efforts to make these older data publicly available.

While we did not perform statistical tests for any of the experimental design elements, differences between animal and human studies seem apparent for sample size, control condition, randomisation, blinding, statistical analyses, sex, age, preceding treatment, MTX resistance, comedication, administration route and analysed outcomes. This suboptimal alignment can be caused on either side; experimenters performing human or animal studies should keep translation in mind when designing their studies. However, there can be good reasons to not always fully align animal and human studies of the same potential treatment. For example, the administration route is preferentially oral for patients needing long-term treatments, or those participating in long-term clinical trials. However, there are two major challenges with oral administration of an investigational drug in preclinical animal studies. First, it is very difficult to administer specific doses to animals with voluntary oral intake via food or water, which is more comparable to human oral administration than direct intragastric administration (gavage). Second, in early phases of drug development, new compounds are not yet available in oral formulations (tablets or palatable liquid). For early preclinical trials of investigational drugs, it may thus be preferable to establish efficacy using alternative administration routes, before starting the sometimes-complicated process of pharmaceutical formulation.

While our study is the first large direct comparison of trial design between animal and human studies, the observed values seem to be in line with preceding work, as discussed for several examples below. An overall problem in (reviewing) scientific literature is the lack of reporting of experimental details [2,3,4,5,6]. The RA-field is no exception, as e.g., clearly shown by an SR of in vivo animal experiments published in the “*Annals of the Rheumatic Diseases*” and “*Arthritis and Rheumatism*” [41]. Recent SRs of human RA studies did not describe percentages of included studies reporting experimental details [42,43], restricting the interpretation of our data in relation to theirs.

In this SR, randomisation was reported in 38% of the animal and 78% of the human studies. In preceding reviews, randomisation was reported in e.g., 17.1% [41], 24,8% [6], 32.4% [44], 36% [2] and 77% [3] of the included animal studies. In our study, we only scored if the authors mentioned randomisation. If the authors did not describe randomisation, they could still have used it, but we are specifically concerned about proper randomisation for animal studies. We still hear scientists state that genetically inbred mice are all the same, while clear behavioural differences are present between the unafraid slow ones that are caught first and the more escape-prone ones that are the last to leave the box. We did not evaluate if the method of randomisation was adequate (e.g., picking an animal from a box is not random) or even possible (one cannot randomise in e.g., observational designs). Of note, a preceding review of animal studies for pancreatitis showed that while 77% of the studies mentioned randomisation, only two of them described the actual method used, and only one provided sufficient details for evaluation [3].

In this SR, at least partial blinding was reported in 13% of the animal and 68% of the human studies. In preceding reviews, it was for 11% [2,44] or 15% [3] of the animal studies. The evaluation of blinding is often complicated by a limited description of the methods used to ensure blinding, and also when (i.e., at which time within the study) the blind was broken. Commonly used generic descriptions such as single-blind or double-blind (as in Figure 5) do not reflect who was blinded to what, for how long and how [45]. The importance of randomisation and blinding to prevent several types of bias (e.g., [46]) is clear for both animal [44,47] and human [48,49] studies.

In this SR, power calculations were reported in 3% of the animal and 26% of the human studies. As our focus for this large review was on the reporting, we cannot conclude anything on the included studies being adequately powered. However, the low level of reporting is concerning, and one would expect to find a proportion of the included animal and human studies to be underpowered as current perverse incentives can encourage scientists to focus on small underpowered studies [50].

Of necessity, we analysed animal age rather crudely. At this stage, it is not clear if ageing can be compared overall between species, or if separate comparisons should be made for different characteristics (e.g., bone closure, sexual maturity, immune status, neurodegeneration, DNA damage and telomere length). We also disregarded strain differences within species, resulting in further imprecision (e.g., for mice, mean life span varies up to 20% with strain and sex [51]). The humanised age values are thus not precise; they should be considered explorative and be interpreted with caution. However, the observed difference between humanised ages for animals and human ages is large and clearly noticeable. Also, the use of relatively young animals is common in other research fields. For example, for animal studies of sepsis, it was estimated that appropriately aged animals were used in less than 1% of the published literature [52].

The main strength of this SR is its size; we performed a full analysis of 659 references. The search date in February 2016 can be seen as a limitation, but we consider the included references to comprise a valid sample for a comparison of trial designs between animal and human studies. Completion of this review took a long time for several reasons. First of all, we screened the titles and abstracts of 8103 references and the full texts of 1405. Second, we extracted the data from 659 references. Third, because we shared the data extraction workload between seven reviewers, cleaning and harmonisation of the data required more time than expected. We strongly recommend avoiding free text data extraction by multiple reviewers in future reviews, e.g., by using drop-down lists, by having one person extract all the data, or, in future, by using automated data extraction.

We have implemented a systematic sampling strategy and compared experimental designs between animal and human studies for a single drug (MTX) used to treat a single disorder (RA). While we observed important differences in the experimental designs, MTX is an effective drug in both animal models and clinical studies [8,53,54]. We could think that translation for MTX in the treatment of RA is good. Note that various definitions of animal-to-human translational success exist, but consensus on how to assess translation is lacking (e.g., [1]). This is only one example, which does not rule out that harmonisation of trial design between animal and human studies can improve translation. The observed heterogeneity in experimental designs in combination with good translation might, however, point to the need of implementing standardised variation in preclinical study designs; if a drug is effective in a multitude of studies using various designs, further extrapolation of the findings, e.g., to another species, could be more likely. In line with this, a preceding narrative review concluded that most approved drugs for RA treatment show therapeutic efficacy in multiple animal models [53]. Furthermore, simulation studies have shown that reproducibility improves with study sample heterogeneity [55].

Animal studies were performed in rats (k = 99), mice (k = 43), both rats and mice (k = 3) and rabbits (k = 2). While these small species might not be suitable to model joint-related diseases in humans because of differences in joint size and cartilage thickness [56], translational success may generally be high in the field of RA. In December 2015, we searched clinical trial registries and medicine compendia to compose a list of compounds that have been administered for RA treatment in human patients. We did not identify any compounds lacking efficacy. It thus seems that the compounds that have progressed to clinical RA trials are indeed effective. Of note, registration of clinical trials only became mandatory in 2007 and data from clinical tests that started before are not all in the public domain. Mandatory clinical trial registration has been a great measure to improve the access to clinical trial data. If voluntary preclinical trial registration for preclinical studies is sufficient to improve the access to preclinical data remains to be seen.

The absence of compounds lacking efficacy in clinical trial registries shows the main limitation of SRs; they can only be used to answer research questions when data are available in the public domain. We created the list of compounds used for experimental and clinical RA treatment because we considered an SR comparing investigational approaches and preclinical trial designs for drugs that were effective and ineffective in RA treatment. Without compounds lacking efficacy, this review was not possible. Furthermore, we considered an SR of the preclinical testing of the new generation of RA drugs (the biologicals) in classic animal models. Our scoping searches did however not identify any studies describing the testing of biologicals in these classic models. This indicates that biologicals were either not tested in classic animal models, or that these tests were not published. Testing of biologicals in a different species than the target species would be expected to induce an adverse immune response, but several of them have been tested in animal models for arthritis since our scoping searches [57,58,59]. However, several developmental drugs against RA seem to have progressed into clinical development without in vivo validation [53].

## 5. Conclusions

We conclude that alignment of experimental designs for animal and human studies is far from optimal. The data described in this paper contribute to the discussion on the value of animal models for pharmaceutical drug development in the 21st century, as misalignment of the experimental designs is expected to decrease external validity of the findings and limit their translational value. However, on their own, these data are insufficient to determine the translational value of animal studies for RA. While it has been stated that the field would be better off if we halted all primary research and expended the same energy in analysing and using the available data [39], other authors have emphasised the usefulness of RA animal models for analysing pathological mechanisms and drug efficacy [53,54]. RA can be studied with several animal-free technologies based on human biology [7]. However, based on the currently available information, we cannot (yet) conclude that all animal experiments can be replaced by alternative methods such as in silico modelling studies and in vitro studies of human materials.

The presented data are specific to the field of RA, and further literature studies are needed to determine if the alignment of animal and human trial design could be better or worse in other research fields. Nevertheless, before proceeding with new animal and clinical studies, further evaluation of the already available evidence-base is warranted to determine what optimal translational strategies should look like. Scientists performing animal and human studies should keep translation in mind and adapt their designs to minimise misalignment. This evaluation will decrease research waste, benefit laboratory animals, human trial participants and eventually human patients. To ensure the maximum advantage of this approach, and to minimise research waste, the availability and reporting quality of the new and older preclinical and clinical data still need to be improved.

## Figures and Tables

**Figure 10:**
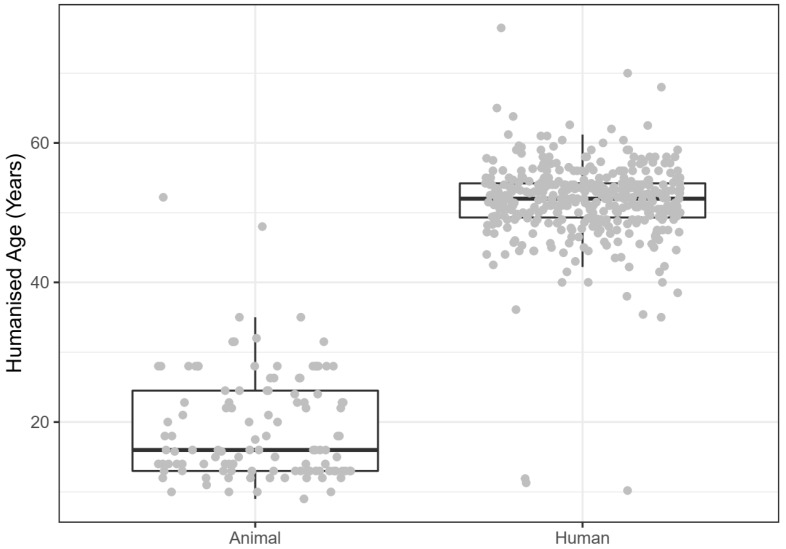
(Humanised) age in years in animal and human studies.

## Supplementary Materials

The following are available online at https://www.mdpi.com/2076-2615/10/6/1047/s1, Table S1: full search strategies, Document S2: list of all included papers.
