# Supplementary material for: A Systematic Review Comparing Experimental Design of Animal and Human Methotrexate Efficacy Studies for Rheumatoid Arthritis: Lessons for the Translational Value of Animal Studies
Source: Animals (Basel). 2020 Jun 17;10(6):1047. doi: 10.3390/ani10061047 (PMC7341304; doi:10.3390/ani10061047)
Supplement: Supplementary file 1 [file animals-10-01047-s001.zip › Search strategies Supplement 1.docx]

**Supplement 1: Search strategies**

| **Database** | **Search Element** | **Search String** |
| --- | --- | --- |
| Pubmed | Rheumatoid Arthritis | Arthritis, Rheumatoid [MeSH] OR Rheumatoid Arthritis [tiab] OR (Rheumatoid [tiab] AND Nodul* [tiab]) OR (Rheumatoid [tiab] AND Vasculiti* [tiab]) OR Arthritis, Experimental [MeSH] OR RA model* [tiab] OR rheumatic arthritis [tiab] OR ((Collagen‐Induced Arthritides [tiab] OR Collagen‐Induced Arthritis [tiab] OR (Arthritides [tiab] AND collagen [tiab]) OR (arthritis[tiab] AND (collagen[tiab] OR (Collagen [tiab] AND antibody [tiab]AND induced [tiab] AND arthritis [tiab]) OR collagens[tiab] OR adjuvant*[tiab] OR experimental[tiab])) OR Arthritides [tiab] OR Collagen type II [MeSH] OR (Type II [tiab] AND (Collagen [tiab] OR Procollagen [tiab] OR Col2a1 [tiab] OR chondrocalcin [tiab])) OR CIA [tiab] OR Proteoglycans [Mesh] OR Proteoglycans [tiab] OR Proteoglycan [tiab] OR PGIA [tiab] OR HSPG [tiab] OR Proteoheparan Sulfate* [tiab] OR glypican* [tiab] OR syndecan* [tiab] OR CD138 Antigens [tiab] OR CD138 Antigen [tiab] OR Fibroglycan [tiab] OR Ryudocan [tiab] OR Amphiglycan [tiab] OR Proteochondroitin Sulfate [tiab] OR Proteochondroitin Sulfates [tiab] OR Aggrecans [tiab] OR Aggrecan [tiab] OR Versicans [tiab] OR Versican [tiab] OR Biglycan [tiab] OR Decorin [tiab] OR DSPG‐II [tiab] OR Hyalectins [tiab] OR Brevican [tiab] OR Neurocan [tiab] OR Lectins, C‐Type [tiab] OR Nerve Tissue Proteins [tiab] OR Citrulline [MeSH] OR Citrul* [tiab] OR Freund's Adjuvant [MeSH] OR adjuvant [tiab] OR Freund* [tiab]) OR Mycobacterium tuberculosis [MeSH] OR Mycobacterium tuberculosis [tiab] OR Mycobacterium butyricum [tiab] OR Antigen induced arthritis [tiab] OR AIA [tiab] OR (Streptococcal [tiab] AND induced [tiab] AND arthritis [tiab]) OR SCW‐A [tiab] OR CAIA [tiab] OR K/BxN model [tiab] OR G6PI‐induced arthritis [tiab] OR SKG [tiab] OR TNF transgenic [tiab] OR gp130 arthritis model [tiab] OR IL‐1 transgenic [tiab] OR pristane induced arthritis [tiab] OR PIA [tiab] OR oil induced arthritis [tiab] OR OIA [tiab]) AND (RA [tiab] OR rheumatism [tiab])) |
|  | Human studies | clinical study [pt] OR clinical trial [MeSH] OR clinical trial [tiab] OR intervention study [tiab] OR controlled clinical trial [MeSH] OR clinical trial as topic [MeSH] OR first in man [tiab] OR proof of concept [tiab] OR randomized controlled trial [pt] OR controlled clinical trial [pt] OR randomized [tiab] OR placebo [tiab] OR drug therapy [sh] OR randomly [tiab] OR trial [tiab] OR groups [tiab] |
|  | Animal studies | SYRCE animal filter (www.SYRCLE.nl) |
|  | Methotrexate | methotrexate [MeSH] OR methotrexate [tiab] OR MTX [tiab] OR Ametopterine [tiab] OR Mexate [tiab] OR Abitrexate [tiab] OR Emtexate [tiab] OR Emthexate [tiab] OR Farmitrexate [tiab] OR Folex [tiab] OR Ledertrexate [tiab] OR Methoblastin [tiab] OR Methohexate [tiab] OR Methotrate [tiab] OR Methylaminopterin [tiab] OR Methotrexate [tiab] OR Novatrex [tiab] OR Rheumatrex [tiab] OR metoject [tiab] OR maxtrex [tiab]  *Note that the term “Amethopterin” should be included for future searches.* |
|  | | |
| Embase | Rheumatoid Arthritis | Rheumatoid arthritis/ OR rheumatoid arthritis.ti,ab,kw. OR ((Type II and (Collagen OR Procollagen or Col2a1 OR chondrocalcin)).ti,ab. OR exp experimental arthritis/ OR exp adjuvant arthritis/ OR exp collagen type 2/ OR exp proteoglycan/ OR exp proteoheparan sulfate/ OR exp aggrecan/ OR exp citrulline/ OR exp freund adjuvant/ OR Glypican$2.ti,ab,kw. OR Syndecan$2.ti,ab,kw. OR CD138 Antigens.ti,ab,kw. OR CD138 Antigen.ti,ab,kw. OR Heparan Sulfate$.ti,ab,kw. OR Chondroitin Sulfate Proteoglycans.ti,ab,kw. OR Chondroitin Sulfate Proteoglycan.ti,ab,kw. OR Proteochondroitin Sulfate.ti,ab,kw. OR Proteochondroitin Sulfates.ti,ab,kw. OR DSPG‐II.ti,ab,kw. OR Lectins, C‐Type.ti,ab,kw. OR Nerve Tissue Proteins.ti,ab,kw. OR Citrul$.ti,ab,kw. OR adjuvant induced arthritis.ti,ab,kw. OR Mycobacterium tuberculosis.ti,ab,kw. OR Mycobacterium tuberculosis H37Rv.ti,ab,kw. OR Mycobacterium butyricum.ti,ab,kw. OR Antigen induced arthritis.ti,ab,kw. OR AIA.ti,ab,kw. OR (Streptococcal AND induced AND arthritis).ti,ab,kw. OR SCW‐A.ti,ab,kw. OR (Collagen AND antibody AND induced AND arthritis).ti,ab,kw. OR BxN model.ti,ab,kw. OR G6PI‐induced arthritis.ti,ab,kw. OR TNF transgenic.ti,ab,kw. OR gp130 arthritis model.ti,ab,kw. OR IL‐1 transgenic.ti,ab,kw. OR pristane induced arthritis.ti,ab,kw. OR oil induced arthritis.ti,ab,kw. OR ((CIA OR PGIA OR HSPG OR Glypicans OR Glypican OR Syndecans OR Syndecan OR Fibroglycan OR Ryudocan OR Amphiglycan OR Aggrecan OR Aggrecans OR Versicans OR Versican OR Biglycan OR Decorin OR Hyalectins OR Brevican OR neurocan OR SKG OR PIA OR OIA OR CAIA).ti,ab,kw.) AND (RA.ti,ab,kw. OR rheumatism.ti,ab,kw.)) |
|  | Human studies | exp clinical trial/ OR clinical study/ OR human subject.ti,ab,kw. OR clinical drug trial.ti,ab,kw. OR major clinical trial.ti,ab,kw. OR trial, clinical.ti,ab,kw. OR clinical study.ti,ab,kw. OR phase 1 clinical trial.ti,ab,kw. OR phase 2 clinical trial.ti,ab,kw. OR phase 3 clinical trial.ti,ab,kw. OR clinical trial, controlled.ti,ab,kw. OR clinical trial, phase 1.ti,ab,kw. OR clinical trial, phase 2.ti,ab,kw. OR clinical trial, phase 3.ti,ab,kw. OR clinical trials.ti,ab,kw. OR clinical trial, phase I.ti,ab,kw. OR clinical trial, phase II.ti,ab,kw. OR clinical trial, phase III.ti,ab,kw. OR intervention study.ti,ab,kw. |
|  | Animal studies | SYRCE animal filters (www.SYRCLE.nl) |
|  | Methotrexate | methotrexate/ OR methotrexate.ti,ab,kw. OR MTX.ti,ab,kw. OR Ametopterine.ti,ab,kw. OR Mexate.ti,ab,kw. OR Abitrexate.ti,ab,kw. OR Emtexate.ti,ab,kw. OR emthexate.ti,ab,kw. OR Farmitrexate.ti,ab,kw. OR Folex.ti,ab,kw. OR Ledertrexate.ti,ab,kw. OR Methoblastin.ti,ab,kw. OR Methohexate.ti,ab,kw. OR Methotrate.ti,ab,kw. OR Methylaminopterin.ti,ab,kw. OR Methotrexate.ti,ab,kw. OR Novatrex.ti,ab,kw. OR Rheumatrex.ti,ab,kw. OR metoject.ti,ab,kw. OR maxtrex.ti,ab,kw.  *Note that the term “Amethopterin” should be included for future searches.* |
