# Supplementary material for: A Systematic Review Comparing Experimental Design of Animal and Human Methotrexate Efficacy Studies for Rheumatoid Arthritis: Lessons for the Translational Value of Animal Studies
Source: Animals (Basel). 2020 Jun 17;10(6):1047. doi: 10.3390/ani10061047 (PMC7341304; doi:10.3390/ani10061047)
Supplement: Supplementary file 1 [file animals-10-01047-s001.zip › Supplement 2 Included papers.pdf]

List of included papers

Study ID	Title	Journal
Abe_1995	Combination therapy on murine arthritis - Salazosulfapyridine, bucillamine, and methotrexate	International_Journal_of_Immunotherapy
Abe_2006	A multicenter, double-blind, randomized, placebo controlled trial of infliximab combined with low dose methotrexate in Japanese patients with rheumatoid arthritis	Journal_of_Rheumatology
Ablin_2010	Protective effect of eotaxin-2 inhibition in adjuvant-induced arthritis	Clinical_Experimental_Immunology
Achira_2002	Decreased activity of hepatic P-glycoprotein in the isolated perfused liver of the adjuvant arthritis rat	Xenobiotica
Agadzhanian_1986	Methotrexate in the treatment of rheumatoid arthritis. [Russian]	Terapevticheskii_arkhiv
Ahmed_2015	Protective Effects of Simvastatin and Hesperidin against Complete Freund's Adjuvant-Induced Rheumatoid Arthritis in Rats	Pharmacology
AlAbd_2010	Nimesulide improves the disease modifying anti-rheumatic profile of methotrexate in mice with collagen-induced arthritis	European_Journal_of_Pharmacology
Alam_2012	Comparative study on methotrexate and hydroxychloroquine in the treatment of rheumatoid arthritis	Mymensingh_medical_journal_:_MMJ
Alarcon_1990	Suppression of rheumatoid factor production by methotrexate in patients with rheumatoid arthritis. Evidence for differential influences of therapy and clinical status on IgM and IgA rheumatoid factor expression	Arthritis_Rheumatism
Alarcon_1992a	Efficacy and safety of 10-deazaaminopterin in the treatment of rheumatoid arthritis: A one-year continuation, double-blind study	Arthritis_Rheumatism
Alarcon_1992b	Controlled trial of methotrexate versus 10-deazaaminopterin in the treatment of rheumatoid arthritis	Ann_Rheum_Dis
Aletaha_2007	Disease activity early in the course of treatment predicts response to therapy after one year in rheumatoid arthritis patients	Arthritis_Rheumatism
Aletaha_2009	Rheumatoid arthritis joint progression in sustained remission is determined by disease activity s preceding the period of radiographic assessment	Arthritis_Rheumatism
Aletaha_2013	Rituximab dissociates the tight link between disease activity and joint damage in rheumatoid arthritis patients	Ann_Rheum_Dis
Ali_1997	Efficacy of methotrexate in rheumatoid arthritis	Bangladesh_Medical_Research_Council_bulletin
Allaart_2006	Aiming at low disease activity in rheumatoid arthritis with initial combination therapy or initial monotherapy strategies: the BeSt study	Clin_Exp_Rheumatol
Alten_2010	Efficacy and safety of pamapimod in patients with active rheumatoid arthritis receiving stable methotrexate therapy	Ann_Rheum_Dis
Andersen_1985	Weekly pulse methotrexate in rheumatoid arthritis. Clinical and immunologic effects in a randomized, double-blind study	Annals_of_internal_medicine
Andersen_1997	Prospectively measured red cell folate s in methotrexate treated patients with rheumatoid arthritis: Relation to withdrawal and side effects	Journal_of_Rheumatology
Andersen_2015	Interleukin-23 in early disease development in rheumatoid arthritis	Scandinavian_Journal_of_Rheumatology

Anderson_1989	Which traditional measures should be used in rheumatoid arthritis clinical trials?	Arthritis_Rheumatism
Anonymus_1995	The effect of age and renal function on the efficacy and toxicity of methotrexate in rheumatoid arthritis. Rheumatoid Arthritis Clinical Trial Archive Group	J_Rheumatol
Asanuma_2002	Weekly pulse therapy of methotrexate improves survival compared with its daily administration in MRL/lpr mice	European_Journal_of_Pharmacology
Atsumi_2016	The first double-blind, randomised, parallel-group certolizumab pegol study in methotrexate-naive early rheumatoid arthritis patients with poor prognostic factors, C-OPERA, shows inhibition of radiographic progression	Ann_Rheum_Dis
Baharav_2012	Immunomodulatory effect of sertraline in a rat model of rheumatoid arthritis	NeuroImmunoModulation
Bakker_2010	Are switches from oral to subcutaneous methotrexate or addition of ciclosporin to methotrexate useful steps in a tight control treatment strategy for rheumatoid arthritis? A post hoc analysis of the CAMERA study	Ann_Rheum_Dis
Bakker_2012	Low-dose prednisone inclusion in a methotrexate-based, tight control strategy for early rheumatoid arthritis: A randomized trial	Annals_of_Internal_Medicine
Banji_2011	Evaluation of the concomitant use of methotrexate and curcumin on Freund's complete adjuvant-induced arthritis and hematological indices in rats	INDn_Journal_of_Pharmacology
Bankhurstk_1999	Etanercept and methotrexate combination therapy	Clinical_Experimental_Rheumatology
Bao_2003	Leflunomide, a new disease-modifying drug for treating active rheumatoid arthritis in methotrexate-controlled phase II clinical trial	Chinese_Medical_Journal
Bao_2011	Secondary failure to treatment with recombinant human IL-1 receptor antagonist in Chinese patients with rheumatoid arthritis	Clinical_Rheumatology
Bao_2014	Good response to infliximab in rheumatoid arthritis following failure of interleukin-1 receptor antagonist	Int_J_Rheum_Dis
Barck_2004	Quantification of cortical bone loss and repair for therapeutic evaluation in collagen-induced arthritis, by micro-computed tomography and automated image analysis	Arthritis_Rheumatism
Barrera_1993	Circulating soluble tumor necrosis factor receptors, interleukin-2 receptors, tumor necrosis factor alpha, and interleukin-6 s in rheumatoid arthritis. Longitudinal evaluation during methotrexate and azathioprine therapy	Arthritis_Rheumatism
Barrera_1994	Circulating concentrations and production of cytokines and soluble receptors in rheumatoid arthritis patients: Effects of a single dose methotrexate	British_Journal_of_Rheumatology
Barrera_1995	Effect of methotrexate alone or in combination with sulphasalazine on the production and circulating concentrations of cytokines and their antagonists. Longitudinal evaluation in patients with rheumatoid arthritis	Br_J_Rheumatol
Barrera_2002	Drug survival, efficacy and toxicity of monotherapy with a fully human anti-tumour necrosis factor-alpha antibody compared with methotrexate in long-standing rheumatoid arthritis	Rheumatology
Barsig_2005	Methotrexate treatment suppresses local cytokine and chemokine production in rat adjuvant arthritis	Drugs_under_Experimental_Clinical_Research
Bathon_2000	A comparison of etanercept and methotrexate in patients with early rheumatoid arthritis	New_England_Journal_of_Medicine

Bathon_2003	The Early Rheumatoid Arthritis (ERA) Trial comparing the efficacy and safety of etanercept and methotrexate	Clinical_Experimental_Rheumatology
Bathon_2006	Safety and efficacy of etanercept treatment in elderly subjects with rheumatoid arthritis	Journal_of_Rheumatology
Bathon_2011	Sustained disease remission and inhibition of radiographic progression in methotrexate-naive patients with rheumatoid arthritis and poor prognostic factors treated with abatacept: 2-Year outcomes	Ann_Rheum_Dis
Bauerova_2010	Combined methotrexate and coenzyme Q<inf>10</inf> therapy in adjuvant-induced arthritis evaluated using parameters of inflammation and oxidative stress	Acta_Biochimica_Polonica
Bauerova_2015	Markers of inflammation and oxidative stress studied in adjuvant-induced arthritis in the rat on systemic and local affected by pinosylvin and methotrexate and their combination	Autoimmunity
Bejarano_2008	Effect of the early use of the anti-tumor necrosis factor adalimumab on the prevention of job loss in patients with early rheumatoid arthritis	Arthritis_Care_Research
Bejarano_2010	Benefits 8 years after a remission induction regime with an infliximab and methotrexate combination in early rheumatoid arthritis	Rheumatology
Bendele_1999a	Effects of interleukin 1 receptor antagonist alone and in combination with methotrexate in adjuvant arthritic rats	Journal_of_Rheumatology
Bendele_1999b	Animal models of arthritis: Relevance to human disease	Toxicologic_Pathology
Bendele_1999c	Effects of PEGylated soluble tumor necrosis factor receptor type I (PEG sTNF-RI) alone and in combination with methotrexate in adjuvant arthritic rats	Clinical_Experimental_Rheumatology
Benenson_1994	Prospidine versus methotrexate pulse in highly active rheumatoid arthritis: A controlled 6-month clinical trial	Clinical_Rheumatology
Bian_2015	[Effect of Combination Therapy of Tetramethylpyrazine with Methotrexate on Inflammatory Reac- tions and Hemorheology in Collagen-induced Arthritis Rats]	Zhongguo_Zhong_Xi_Yi_Jie_He_Za_Zhi
Biasi_1996	Efficacy and safety of low dose methotrexate in elderly onset rheumatoid arthritis [5]	Journal_of_Rheumatology
Bilasy_2015	Myelosuppressive and hepatotoxic potential of leflunomide and methotrexate combination in a rat model of rheumatoid arthritis	Pharmacological_Reports
Bingham_2006	Is combined etanercept and methotrexate more efficacious than either monotherapy for treating RA?: Commentary	Nature_Clinical_Practice_Rheumatology
Bingham_2014	The effect of intravenous golimumab on health-related quality of life in rheumatoid arthritis: 24-week results of the phase III GO-FURTHER trial	J_Rheumatol
Boechat_2015	Methotrexate-loaded lipid-core nanocapsules are highly effective in the control of inflammation in synovial cells and a chronic arthritis model	International_Journal_of_Nanomedicine
Bologna_1995	Correlation between methotrexate pharmacokinetic parameters, and clinical and biological status in rheumatoid arthritis patients	Clinical_Experimental_Rheumatology
Boyle_2015	The JAK inhibitor tofacitinib suppresses synovial JAK1-STAT signalling in rheumatoid arthritis	Ann_Rheum_Dis
Brahn_1991	Suppression of collagen-induced arthritis by combination cyclosporin A and methotrexate therapy	Arthritis_Rheumatism

Braun_2008	Comparison of the clinical efficacy and safety of subcutaneous versus oral administration of methotrexate in patients with active rheumatoid arthritis: Results of a six-month, multicenter, randomized, double-blind, controlled, phase IV trial	Arthritis_Rheumatism
Breedveld_2004	Infliximab in active early rheumatoid arthritis	Ann_Rheum_Dis
Breedveld_2006	The PREMIER study: A multicenter, randomized, double-blind clinical trial of combination therapy with adalimumab plus methotrexate versus methotrexate alone or adalimumab alone in patients with early, aggressive rheumatoid arthritis who had not had previous methotrexate treatment	Arthritis_Rheumatism
Breedveld_2007	Rituximab pharmacokinetics in patients with rheumatoid arthritis: B-cell s do not correlate with clinical response	Journal_of_Clinical_Pharmacology
Breshnihan_2001	Treating early rheumatoid arthritis in the younger patient	J_Rheumatol_Suppl
Bresnihan_2002	Serum interleukin 18 and interleukin 18 binding protein in rheumatoid arthritis	Ann_Rheum_Dis
Bruyn_2008	Everolimus in patients with rheumatoid arthritis receiving concomitant methotrexate: A 3-month, double-blind, randomised, placebo-controlled, parallel-group, proof-of-concept study	Ann_Rheum_Dis
Buckley_1997	Effects of low dose methotrexate on the bone mineral density of patients with rheumatoid arthritis	Journal_of_Rheumatology
Buhroo_2000	Observations on longterm results of methotrexate in rheumatoid arthritis patients in Kashmir	JK_Practitioner
Burmester_2011	Mavrilimumab, a human monoclonal antibody targeting GM-CSF receptor-alpha, in subjects with rheumatoid arthritis: A randomised, double-blind, placebo-controlled, phase I, first-in-human study	Ann_Rheum_Dis
Burmester_2013a	Tofacitinib (CP-690,550) in combination with methotrexate in patients with active rheumatoid arthritis with an inadequate response to tumour necrosis factor inhibitors: A randomised phase 3 trial	Lancet
Burmester_2013b	Efficacy and safety of mavrilimumab in subjects with rheumatoid arthritis	Ann_Rheum_Dis
Burmester_2015	Tocilizumab in early progressive rheumatoid arthritis: FUNCTION, a randomised controlled trial	Ann_Rheum_Dis
Capell_2006	Combination therapy with sulfasalazine and methotrexate is more effective than either drug alone in patients with rheumatoid arthritis with a suboptimal response to sulfasalazine: Results from the double-blind placebo-controlled MASCOT study	Ann_Rheum_Dis
Capone_2000	Are there differences in methotrexate kinetics between responding and nonresponding patients with rheumatoid arthritis?	BioDrugs
Cardiel_2010	A phase 2 randomized, double-blind study of AMG 108, a fully human monoclonal antibody to IL-1R, in patients with rheumatoid arthritis	Arthritis_Res_Ther
Carvallo_1993	[Rheumatoid arthritis. Therapeutic efficacy of methotrexate and its hepatotoxic effects]	Rev_Med_Chil
Castaneda_2006	Controlled trial of methotrexate versus CH-1504 in the treatment of rheumatoid arthritis	Journal_of_Rheumatology
CharlesSchoeman_2015	Association of Triple Therapy with Improvement in Cholesterol Profiles over Two Year Follow-up in the TEAR Trial	Arthritis_Rheumatol
Chen_2005	Tongbiling for interleukin 2 and its receptor alpha-chain in vivo and in vitro	Chinese_Journal_of_Clinical_Rehabilitation

Chen_2009	Randomized, double-blind, placebo-controlled, comparative study of human anti-TNF antibody adalimumab in combination with methotrexate and methotrexate alone in Taiwanese patients with active rheumatoid arthritis	Journal_of_the_Formosan_Medical_Association
Chen_2010	Efficacy of Shenshi Qianghuo Dihuang Decoction in rheumatoid arthritis: A randomized controlled trial. [Chinese]	Journal_of_Chinese_Integrative_Medicine
Chen_2012	Prediction of response of collagen-induced arthritis rats to methotrexate: An 1H-NMR-based urine metabolomic analysis	Journal_of_Huazhong_University_of_Science_Technology_-_Medical_Science
Chen_2013	A multicenter, randomized, double-blind clinical trial of combination therapy with Anbainuo, a novel recombinant human TNFRII:Fc fusion protein, plus methotrexate versus methotrexate alone or Anbainuo alone in Chinese patients with moderate to severe rheumatoid arthritis	Clinical_Rheumatology
Chen_2015	Meridian-sinew release therapy for the treatment of refractory rheumatoid arthritis	International_Journal_of_Clinical_Experimental_Medicine
Cheng_2014	Tartrate-resistant acid phosphatase 5b is a potential biomarker for rheumatoid arthritis: a pilot study in Han Chinese	Chin_Med_J_(Engl)
Cherie_1995	Low dose methotrexate in the treatment of rheumatoid arthritis and psoriatic arthropathy. [Italian]	Rivista_Italiana_di_Biologia_e_Medicina
Chopra_2015	Itolizumab in combination with methotrexate modulates active rheumatoid arthritis: safety and efficacy from a phase 2, randomized, open-label, parallel-group, dose-ranging study	Clin_Rheumatol
Cohen_2001	Two-year, blinded, randomized, controlled trial of treatment of active rheumatoid arthritis with leflunomide compared with methotrexate. Utilization of Leflunomide in the Treatment of Rheumatoid Arthritis Trial Investigator Group	Arthritis_Rheumatism
Cohen_2002	Treatment of rheumatoid arthritis with anakinra, a recombinant human interleukin-1 receptor antagonist, in combination with methotrexate: Results of a twenty-four-week, multicenter, randomized, double-blind, placebo-controlled trial	Arthritis_Rheumatism
Cohen_2003	Interleukin 1 receptor antagonist anakinra improves functional status in patients with rheumatoid arthritis	Journal_of_Rheumatology
Cohen_2004	A multicentre, double blind, randomised, placebo controlled trial of anakinra (Kineret), a recombinant interleukin 1 receptor antagonist, in patients with rheumatoid arthritis treated with background methotrexate	Ann_Rheum_Dis
Cohen_2006	Rituximab for rheumatoid arthritis refractory to anti-tumor necrosis factor therapy: Results of a multicenter, randomized, double-blind, placebo-controlled, phase III trial evaluating primary efficacy and safety at twenty-four weeks	Arthritis_Rheumatism
Cohen_2008	Denosumab treatment effects on structural damage, bone mineral density, and bone turnover in rheumatoid arthritis: A twelve-month, multicenter, randomized, double-blind, placebo-controlled, phase II clinical trial	Arthritis_Rheumatism
Cohen_2009	Evaluation of the efficacy and safety of pamapimod, a p38 MAP kinase inhibitor, in a double-blind, methotrexate-controlled study of patients with active rheumatoid arthritis	Arthritis_Rheumatism
Cohen_2010	Continued inhibition of structural damage over 2 years in patients with rheumatoid arthritis treated with rituximab in combination with methotrexate	Ann_Rheum_Dis

Conaghan_2003	Elucidation of the relationship between synovitis and bone damage: A randomized magnetic resonance imaging study of individual joints in patients with early rheumatoid arthritis	Arthritis_Rheumatism
Conaghan_2011	Assessment by MRI of inflammation and damage in rheumatoid arthritis patients with methotrexate inadequate response receiving golimumab: Results of the GO-FORWARD trial	Ann_Rheum_Dis
Connolly_1988	Alteration of interleukin-1 production and the acute phase response following medication of adjuvant arthritic rats with cyclosporin-A or methotrexate	Int_J_Immunopharmacol
Cranney_2001	The effect of low dose methotrexate on bone density	J_Rheumatol
Crawford_2007	Methotrexate in early inflammatory arthritis	Current_Rheumatology_Reports
Crilly_1995	Interleukin 6 (IL-6) and soluble IL-2 receptor s in patients with rheumatoid arthritis treated with low dose oral methotrexate	Journal_of_Rheumatology
Currey_1971	A comparison of immunosuppressive and antiinflammatory agents in the rat	Rhumatologie_-_Revue_International_de_Rhumatologie
Cuzzocrea_2005	Artemether: A new therapeutic strategy in experimental rheumatoid arthritis	Immunopharmacology_Immunotoxicology
Damjanov_2009	Efficacy, pharmacodynamics, and safety of VX-702, a novel p38 MAPK inhibitor, in rheumatoid arthritis: Results of two randomized, double-blind, placebo-controlled clinical studies	Arthritis_Rheumatism
Danquah_2011	Anti-arthritic effects of an ethanolic extract of capparis erythrocarpos isert roots in freund's adjuvant-induced arthritis in rats	Journal_of_Pharmacology_Toxicology
Datta_2013	Evaluation of anti-arthritic property of methotrexate conjugated gold nanoparticle on experimental animal models	Journal_of_Nanopharmaceutics_Drug_Delivery
DeFang_2015	[Effect of Sanhuang Yilong Decoction combined MTX on the expression of serum IL-1, IL-6, and IL-17 in rheumatoid arthritis patients of accumulated dampness-heat syndrome]	Zhongguo_Zhong_Xi_Yi_Jie_He_Za_Zhi
DeGraaf_1994	Effects of low dose methotrexate therapy on the concentration and the glycosylation of alpha<inf>1</inf>-acid glycoprotein in the serum of patients with rheumatoid arthritis: A longitudinal study	Journal_of_Rheumatology
DeGraw_1995	New analogs of methotrexate in cancer and arthritis	Current_Medicinal_Chemistry
Dejaco_2008	Effect of interleukin-6 receptor inhibition with tocilizumab in patients with rheumatoid arthritis (OPTION study): A double-blind, placebo-controlled, randomised trial. [German]	Journal_fur_Mineralstoffwechsel
Delano_2005	Genetically based resistance to the antiinflammatory effects of methotrexate in the air-pouch model of acute inflammation	Arthritis_Rheumatism
Dell_1996	Treatment of rheumatoid arthritis with methotrexate alone, sulfasalazine and hydroxychloroquine, or a combination of all three medications	New_England_Journal_of_Medicine
Dell_1999	Combination DMARD therapy with hydroxychloroquine, sulfasalazine, and methotrexate	Clinical_Experimental_Rheumatology
Dell_2013	Validation of the methotrexate-first strategy in patients with early, poor-prognosis rheumatoid arthritis: Results from a two-year randomized, double-blind trial	Arthritis_Rheumatism
Deodhar_2010	Denosumab-mediated increase in hand bone mineral density associated with decreased progression of bone erosion in rheumatoid arthritis patients	Arthritis_Care_Research
Dervieux_2012	Patterns of interaction between genetic and nongenetic attributes and methotrexate efficacy in rheumatoid arthritis	Pharmacogenet_Genomics

Desoky_1997	Disposition and clinical efficacy of methotrexate in patients with rheumatoid arthritis following weekly, low, intramuscular dosing: A pilot study	Current_Therapeutic_Research_-_Clinical_Experimental
Detert_2013	Induction therapy with adalimumab plus methotrexate for 24 weeks followed by methotrexate monotherapy up to week 48 versus methotrexate therapy alone for DMARD-naive patients with early rheumatoid arthritis: HIT HARD, aninvestigator-initiated study	Ann_Rheum_Dis
Dhir_2014	Randomized controlled trial comparing 2 different starting doses of methotrexate in rheumatoid arthritis	Clin_Ther
Dominguez_2013a	Therapeutic effect of two altered peptide ligands derived from the human heat shock protein 60 in experimental models of rheumatoid arthritis	Biotecnologia_Aplicada
Dougados_1999	Combination therapy in early rheumatoid arthritis: A randomised, controlled, double blind 52 week clinical trial of sulphasalazine and methotrexate compared with the single components	Ann_Rheum_Dis
Dougados_2009	Evaluation of different methods used to assess disease activity in rheumatoid arthritis: Analyses of Abatacept clinical trial data	Ann_Rheum_Dis
Dougados_2014	When to adjust therapy in patients with rheumatoid arthritis after initiation of etanercept plus methotrexate or methotrexate alone: findings from a randomized study (COMET)	J_Rheumatol
Doyle_2009	Treatment with Infliximab plus Methotrexate Improves Anemia in Patients with Rheumatoid Arthritis Independent of Improvement in Other Clinical Outcome Measures-A Pooled Analysis from Three Large, Multicenter, Double-Blind, Randomized Clinical Trials	Seminars_in_Arthritis_Rheumatism
Drafi_2012	Pharmacological influence on processes of adjuvant arthritis: Effect of the combination of an antioxidant active substance with methotrexate	Interdisciplinary_Toxicology
Drosos_1990	Methotrexate therapy in rheumatoid arthritis. A two year prospective follow-up	Clin_Rheumatol
Drosos_1997	Influence of methotrexate on radiographic progression in rheumatoid arthritis: A sixty-month prospective study	Clinical_Experimental_Rheumatology
Du_2005	Comparative study on clinical efficacy of using methotrexate singly or combined with total glucosides of Paeony in treating rheumatoid arthritis. [Chinese]	Zhongguo_Zhong_Xi_Yi_Jie_He_Za_Zhi
Duan_2015	Efficacy and safety evaluation of a combination of iguratimod and methotrexate therapy for active rheumatoid arthritis patients: a randomized controlled trial	Clin_Rheumatol
Ducreux_2014	Global molecular effects of tocilizumab therapy in rheumatoid arthritis synovium	Arthritis_Rheumatology
Durez_2007	Treatment of early rheumatoid arthritis: A randomized magnetic resonance imaging study comparing the effects of methotrexate alone, methotrexate in combination with infliximab, and methotrexate in combination with intravenous pulse methylprednisolone	Arthritis_Rheumatism
EckerSchlipf_2009	Rheumatoid arthritis: Methotrexate plus etanercept is more effective than monotherapy. [German]	Medizinische_Monatsschrift_fur_Pharmazeuten
Edwards_2004	Efficacy of B-cell-targeted therapy with rituximab in patients with rheumatoid arthritis	New_England_Journal_of_Medicine
Ellingsen_2007a	Differential effect of methotrexate on the increased CCR2 density on circulating CD4 T lymphocytes and monocytes in active chronic rheumatoid arthritis, with a down regulation only on monocytes in responders	Ann_Rheum_Dis

Ellingsen_2007b	In active chronic rheumatoid arthritis, dipeptidyl peptidase IV density is increased on monocytes and CD4(+) T lymphocytes	Scandinavian_Journal_of_Immunology
Ellingsen_2012	Up-regulated dipeptidyl-peptidase IV (CD26) on monocytes was unaffected by effective DMARD treatment in early steroid and DMARD-naive rheumatoid arthritis	Clinical_Experimental_Rheumatology
Ellingsen_2014	Upregulated baseline plasma CCL19 and CCR7 cell-surface expression on monocytes in early rheumatoid arthritis normalized during treatment and CCL19 correlated with radiographic progression	Scandinavian_Journal_of_Rheumatology
ElMiedany_1998	Effect of low dose methotrexate on markers of bone metabolism in patients with rheumatoid arthritis	J_Rheumatol
Emery_1999	Disease modification in rheumatoid arthritis with leflunomide	Scandinavian_Journal_of_Rheumatology
Emery_2000	A comparison of the efficacy and safety of leflunomide and methotrexate for the treatment of rheumatoid arthritis	Rheumatology
Emery_2006a	The efficacy and safety of rituximab in patients with active rheumatoid arthritis despite methotrexate treatment: Results of a phase IIb randomized, double-blind, placebo-controlled, dose-ranging trial	Arthritis_Rheumatism
Emery_2006b	Treatment of rheumatoid arthritis patients with abatacept and methotrexate significantly improved health-related quality of life	Journal_of_Rheumatology
Emery_2008	Comparison of methotrexate monotherapy with a combination of methotrexate and etanercept in active, early, moderate to severe rheumatoid arthritis (COMET): a randomised, double-blind, parallel treatment trial	Lancet
Emery_2009a	Golimumab, a human anti-tumor necrosis factor alpha monoclonal antibody, injected subcutaneously every four weeks in methotrexate-naive patients with active rheumatoid arthritis: Twenty-four-week results of a phase III, multicenter, randomized, double-blind, placebo-controlled study of golimumab before methotrexate as first-line therapy for early-onset rheumatoid arthritis	Arthritis_Rheumatism
Emery_2009b	Less radiographic progression with adalimumab plus methotrexate versus methotrexate monotherapy across the spectrum of clinical response in early rheumatoid arthritis	Journal_of_Rheumatology
Emery_2010a	Two-year clinical and radiographic results with combination etanercept-methotrexate therapy versus monotherapy in early rheumatoid arthritis: A two-year, double-blind, randomized study	Arthritis_Rheumatism
Emery_2010b	Efficacy and safety of different doses and retreatment of rituximab: A randomised, placebo-controlled trial in patients who are biological naive with active rheumatoid arthritis and an inadequate response to methotrexate (Study Evaluating Rituximab's Efficacy in MTX iNadequate rEsponders (SERENE))	Ann_Rheum_Dis
Emery_2011a	Exploratory analyses of the association of MRI with clinical, laboratory and radiographic findings in patients with rheumatoid arthritis	Ann_Rheum_Dis
Emery_2011b	The effects of golimumab on radiographic progression in rheumatoid arthritis: results of randomized controlled studies of golimumab before methotrexate therapy and golimumab after methotrexate therapy	Arthritis_Rheumatism

Emery_2012	Combination etanercept and methotrexate provides better disease control in very early (<4 months) versus early rheumatoid arthritis (>4 months and <2 years): Post hoc analyses from the COMET study	Ann_Rheum_Dis
Emery_2013	Golimumab, a human anti-tumor necrosis factor monoclonal antibody, injected subcutaneously every 4 weeks in patients with active rheumatoid arthritis who had never taken methotrexate: 1-year and 2-year clinical, radiologic, and physical function findings of a phase III, multicenter, randomized, double-blind, placebo-controlled study	Arthritis_Care_Research
Emery_2014	Sustained remission with etanercept tapering in early rheumatoid arthritis	New_England_Journal_of_Medicine
Emery_2014a	Efficacy of golimumab plus methotrexate in methotrexate-naive patients with severe active rheumatoid arthritis	Clin_Rheumatol
Emery_2015b	Evaluating drug-free remission with abatacept in early rheumatoid arthritis: Results from the phase 3b, multicentre, randomised, active-controlled AVERT study of 24 months, with a 12-month, double-blind treatment period	Ann_Rheum_Dis
Emery_2016	Efficacy and safety of subcutaneous golimumab in methotrexate-naive patients with rheumatoid arthritis: 5-year results of the GO-BEFORE trial	Arthritis_Care_Research
Endale_2013	Torilin ameliorates type II collagen-induced arthritis in mouse model of rheumatoid arthritis	International_Immunopharmacology
Fahmy_2015	Ramipril and haloperidol as promising approaches in managing rheumatoid arthritis in rats	European_Journal_of_Pharmacology
Faisal_2015a	Comparative evaluation of thymoquinone and methotrexate in lung inflammation in murine model of rheumatiod arthritis	Journal_of_Postgraduate_Medical_Institute
Faisal_2015b	Comparison of the therapeutic effects of thymoquinone and methotrexate on renal injury in pristane induced arthritis in rats	Journal_of_the_College_of_Physicians_Surgeons_Pakistan
Faisal_2015c	Anti inflammatory effect of thymoquinone in comparison with methotrexate on pristane induced arthritis in rats	Journal_of_the_Pakistan_Medical_Association
Fathi_2002	Longitudinal measurement of methotrexate liver concentrations does not correlate with liver damage, clinical efficacy, or toxicity during a 3.5 year double blind study in rheumatoid arthritis	Journal_of_Rheumatology
Fautrel_2015	Identifying patients with rheumatoid arthritis with moderate disease activity at risk of significant radiographic progression despite methotrexate treatment	RMD_Open
Feketeova_2012	Effect of methotrexate on inflammatory cells redistribution in experimental adjuvant arthritis	Rheumatology_International
Ferraccioli_2002	Analysis of improvements, full responses, remission and toxicity in rheumatoid patients treated with step-up combination therapy (methotrexate, cyclosporin A, sulphasalazine) or monotherapy for three years	Rheumatology
Ferrante_2009	Long-term anti-tumour necrosis factor therapy reverses the progression of carotid intima-media thickness in female patients with active rheumatoid arthritis	Rheumatol_Int
Fiehn_2004a	Methotrexate (MTX) and albumin coupled with MTX (MTX-HSA) suppress synovial fibroblast invasion and cartilage degradation in vivo	Ann_Rheum_Dis
Fiehn_2004b	Albumin-coupled methotrexate (MTX-HSA) is a new anti-arthritic drug which acts synergistically to MTX	Rheumatology

Fiehn_2008	Targeted drug delivery by in vivo coupling to endogenous albumin: An albumin-binding prodrug of methotrexate (MTX) is better than MTX in the treatment of murine collagen-induced arthritis	Ann_Rheum_Dis
Fitzpatrick_2011	Attenuation of arthritis in rodents by a novel orally-available inhibitor of sphingosine kinase	Inflammopharmacology
Fleischhaker_2012	Maraviroc, a chemokine receptor-5 antagonist, fails to demonstrate efficacy in the treatment of patients with rheumatoid arthritis in a randomized, double-blind placebo-controlled trial	Arthritis_Res_Ther
Fleischmann_2013	Tocilizumab inhibits structural joint damage and improves physical function in patients with rheumatoid arthritis and inadequate responses to methotrexate: LITHE study 2-year results	Journal_of_Rheumatology
Franck_1994	Osteocalcin in patients with rheumatoid arthritis. A one-year followup study	Journal_of_Rheumatology
Fransen_2001	Responsiveness of the self-assessed rheumatoid arthritis disease activity index to a flare of disease activity	Arthritis_Rheumatism
Fransen_2004	Influence of guideline adherence on outcome in a randomised controlled trial on the efficacy of methotrexate with folate supplementation in rheumatoid arthritis	Ann_Rheum_Dis
Fransen_2010	Validity of the disease activity score in undifferentiated arthritis	Arthritis_Care_Research
Furst_2011	Double-blind, randomized, controlled, pilot study comparing classic ayurvedic medicine, methotrexate, and their combination in rheumatoid arthritis	J_Clin_Rheumatol
Furst_2015	Two dosing regimens of certolizumab pegol in patients with active rheumatoid arthritis	Arthritis_Care_Research
FuruzawaCarballeda_2012	Polymerized-type i collagen induces upregulation of Foxp3-expressing CD4 regulatory T cells and downregulation of IL-17-producing CD4⁺ T cells (Th17) cells in collagen-induced arthritis	Clinical_Developmental_Immunology
Ganesan_2016	Majoon ushba, a polyherbal compound ameliorates rheumatoid arthritis via regulating inflammatory and bone remodeling markers in rats	Cytokine
Gao_1998	Inhibition of interleukin-8 synthesis by intraarticular methotrexate therapy in patients with rheumatoid arthritis	Z_Rheumatol
Gao_2002	A randomized controlled clinical trial of actarit in the treatment of rheumatoid arthritis. [Chinese]	Chinese_Pharmaceutical_Journal
Gao_2010	[Therapeutic effect of infliximab on moderate and severe active rheumatoid arthritis]. [Chinese]	Nan_fang_yi_ke_da_xue_xue_bao_=_Journal_of_Southern_Medical_University
Gao_2015	Changes in focal adhesion kinase expression in rats with collagen-induced arthritis and efficacy of intervention with disease modifying anti-rheumatic drugs alone or in combination	Int_J_Clin_Exp_Pathol
Garnero_2002	Association of baseline s of urinary glucosyl-galactosyl-pyridinoline and type II collagen C-telopeptide with progression of joint destruction in patients with early rheumatoid arthritis	Arthritis_Rheumatism
Garnero_2002	Association of baseline s of markers of bone and cartilage degradation with long-term progression of joint damage in patients with early rheumatoid arthritis: The COBRA study	Arthritis_Rheumatism
Garnero_2010	Rapid and sustained improvement in bone and cartilage turnover markers with the anti-interleukin-6 receptor inhibitor tocilizumab plus methotrexate in rheumatoid arthritis patients with an inadequate response to methotrexate: Results from a substudy of the multicenter double-blind, placebo-controlled trial of tocilizumab in inadequate responders to methotrexate alone	Arthritis_Rheumatism
Garrison_1999	Etanercept: Therapeutic use in patients with rheumatoid arthritis	Ann_Rheum_Dis

Gatica_2011	Effects of methotrexate on the expression of the translational isoforms of glucocorticoid receptors alpha and beta: correlation with methotrexate efficacy in rheumatoid arthritis patients	Rheumatology
Ge_1989	[Methotrexate (MTX) in the treatment of rheumatoid arthritis]	Zhonghua_Nei_Ke_Za_Zhi
Genovese_2002	Etanercept versus methotrexate in patients with early rheumatoid arthritis: Two-year radiographic and clinical outcomes	Arthritis_Rheumatism
Genovese_2008	Ocrelizumab, a humanized anti-CD20 monoclonal antibody, in the treatment of patients with rheumatoid arthritis: A phase I/II randomized, blinded, placebo-controlled, dose-ranging study	Arthritis_Rheumatism
Genovese_2012	Effect of golimumab on patient-reported outcomes in rheumatoid arthritis: Results from the GO-FORWARD study	Journal_of_Rheumatology
Genovese_2013a	A phase 2 dose-ranging study of subcutaneous tabalumab for the treatment of patients with active rheumatoid arthritis and an inadequate response to methotrexate	Ann_Rheum_Dis
Genovese_2013b	Tabalumab in rheumatoid arthritis patients with an inadequate response to methotrexate and naive to biologic therapy: A phase II, randomized, placebo-controlled trial	Arthritis_Rheumatism
Genovese_2014	A phase III, multicenter, randomized, double-blind, placebo-controlled, parallel-group study of 2 dosing regimens of fostamatinib in patients with rheumatoid arthritis with an inadequate response to a tumor necrosis factor-alpha antagonist	Journal_of_Rheumatology
Genovese_2015	Sarilumab Plus Methotrexate in Patients With Active Rheumatoid Arthritis and Inadequate Response to Methotrexate: Results of a Phase III Study	Arthritis_Rheumatism
Gerlag_2010	Preclinical and clinical investigation of a CCR5 antagonist, AZD5672, in patients with rheumatoid arthritis receiving methotrexate	Arthritis_Rheumatism
Gheorghe_2012	Limited effect of anti-rheumatic treatment on 15-prostaglandin dehydrogenase in rheumatoid arthritis synovial tissue	Arthritis_Res_Ther
Gottschalk_2015	Therapeutic effect of methotrexate encapsulated in cationic liposomes (EndoMTX) in comparison to free methotrexate in an antigen-induced arthritis study in vivo	Scandinavian_Journal_of_Rheumatology
Greisen_2015	Macrophage activity assessed by soluble CD163 in early rheumatoid arthritis: association with disease activity but different response patterns to synthetic and biologic DMARDs	Clin_Exp_Rheumatol
Gubar_2008	Comparison of efficacy and tolerability of triple combination therapy (methotrexate+sulfasalazine+hydroxychloroquine) with methotrexate monotherapy in patients with rheumatoid arthritis. [Russian]	Terapevticheskii_Arkhiv
Gupta_2014	Evaluation of anti-inflammatory effect of Withania somnifera root on collagen-induced arthritis in rats	Pharmaceutical_Biology
Haagsma_1994	Combination of methotrexate and sulphasalazine vs methotrexate alone: A randomized open clinical trial in rheumatoid arthritis patients resistant to sulphasalazine therapy	British_Journal_of_Rheumatology
Haagsma_1995	Combining sulphasalazine and methotrexate in rheumatoid arthritis: Early clinical impressions	British_Journal_of_Rheumatology
Haagsma_1997	Combination of sulphasalazine and methotrexate versus the single components in early rheumatoid arthritis: A randomized, controlled, double-blind, 52 week clinical trial	British_Journal_of_Rheumatology
Haagsma_1999	Influence of sulphasalazine, methotrexate, and the combination of both on plasma homocysteine concentrations in patients with rheumatoid arthritis	Ann_Rheum_Dis

Hamilton_2001	Comparative study of intramuscular gold and methotrexate in a rheumatoid arthritis population from a socially deprived area	Ann_Rheum_Dis
Han_2012	[Effect of fire-needle intervention on serum IL-1 and TNF-alpha s of rheumatoid arthritis rats]. [Chinese]	Zhen_ci_yan_jiu_=_Acupuncture_research___[Zhongguo_yi_xue_ke_xue_yuan_Yi_xue_qing_bao_yan_jiu_suo_bian_ji]
Hang_2005	Double-blind randomized placebo controlled trial (phase II) on the efficacy and safety of Ranjiangduoji capsule in the treatment of patients with active rheumatoid arthritis (Rtention of Cold-damp in the collaterals). [Chinese]	Chinese_Journal_of_Evidence-Based_Medicine
Hanyu_1999	Long-term methotrexate (MTX) combination therapy versus MTX alone for active rheumatoid arthritis	Japanese_Journal_of_Rheumatology
Haroon_2008	A novel predictor of clinical response to methotrexate in patients with rheumatoid arthritis: a pilot study of in vitro T cell cytokine suppression	J_Rheumatol
Hartog_2009	Locomotion and muscle mass measures in a murine model of collagen-induced arthritis	BMC_Musculoskeletal_Disorders
Hashiramoto_2009	Prospective study of methotrexate treatment for rheumatoid arthritis treated legitimately according to the government recommended 8 mg/week dose	Modern_Rheumatology
Haugeberg_2009	Bone loss in patients with active early rheumatoid arthritis: Infliximab and methotrexate compared with methotrexate treatment alone. Explorative analysis from a 12-month randomised, double-blind, placebo-controlled study	Ann_Rheum_Dis
Hauselmann_1998	Can collagen type II sustain a methotrexate-induced therapeutic effect in patients with long-standing rheumatoid arthritis? A double-blind, randomized trial	British_Journal_of_Rheumatology
He_2002	Influence of bizhongxiao (bzx) decoction on the plasma tnf-alpha s of C II-induced rheumatoid arthritis in rats. [Chinese]	Bulletin_of_Hunan_Medical_University
Herborn_1992	Interim report on 102 patients after two years in a double blind comparison of intramuscular methotrexate and gold sodium thiomalate in early erosive rheumatoid arthritis. [German]	Zeitschrift_fur_Rheumatologie
Hetland_2006	Combination treatment with methotrexate, cyclosporine, and intraarticular betamethasone compared with methotrexate and intraarticular betamethasone in early active rheumatoid arthritis: An investigator-initiated, multicenter, randomized, double-blind, parallel-group, placebo-controlled study	Arthritis_Rheumatism
Hetland_2008	Aggressive combination therapy with intra-articular glucocorticoid injections and conventional disease-modifying anti-rheumatic drugs in early rheumatoid arthritis: Second-year clinical and radiographic results from the CIMESTRA study	Ann_Rheum_Dis
Hirata_2015	Serum 14-3-3eta is associated with severity and clinical outcomes of rheumatoid arthritis, and its pretreatment is predictive of DAS28 remission with tocilizumab	Arthritis_Res_Ther
Hisadome_2004	Combination benefit of a pyrimidylpiperazine derivative (Y-40138) and methotrexate in arthritic rats	European_Journal_of_Pharmacology
HjeltnesI_2013	Serum s of lipoprotein(a) and E-selectin are reduced in rheumatoid arthritis patients treated with methotrexate or methotrexate in combination with TNF-alpha-inhibitor	Clinical_Experimental_Rheumatology

Hobl_2011	The influence of methotrexate on the gene expression of the pro-inflammatory cytokine IL-12A in the therapy of rheumatoid arthritis	Clinical_Experimental_Rheumatology
Hobl_2012a	A short-chain methotrexate polyglutamate as outcome parameter in rheumatoid arthritis patients receiving methotrexate	Clinical_Experimental_Rheumatology
Hobl_2012b	A Randomized, Double-Blind, Parallel, Single-Site Pilot Trial to Compare Two Different Starting Doses of Methotrexate in Methotrexate-Naive Adult Patients with Rheumatoid Arthritis	Clinical_Therapeutics
Hoekstra_2003	Factors associated with toxicity, final dose, and efficacy of methotrexate in patients with rheumatoid arthritis	Ann_Rheum_Dis
Hoff_2009	Adalimumab therapy reduces hand bone loss in early rheumatoid arthritis: Explorative analyses from the PREMIER study	Ann_Rheum_Dis
Hoff_2011	Adalimumab reduces hand bone loss in rheumatoid arthritis independent of clinical response: Subanalysis of the PREMIER study	BMC_Musculoskeletal_Disorders
Hornung_2004	Folate, homocysteine, and cobalamin status in patients with rheumatoid arthritis treated with methotrexate, and the effect of low dose folic acid supplement	J_Rheumatol
HorslevPetersen_2014	Adalimumab added to a treat-to-target strategy with methotrexate and intra-articular triamcinolone in early rheumatoid arthritis increased remission rates, function and quality of life. The OPERA Study: An investigator-initiated, randomised, double-blind, parallel-group, placebo-controlled Trial	Ann_Rheum_Dis
Hu_2001	A randomized, controlled, single-blind trial of leflunomide in the treatment of rheumatoid arthritis	Journal_of_Tongji_Medical_University_=_Tong_ji_yi_ke_da_xue_xue_bao
Hu_2009	A comparison study of a recombinant tumor necrosis factor receptor:Fc fusion protein (rhTNFR:Fc) and methotrexate in treatment of patients with active rheumatoid arthritis in China	Rheumatology_International
Huang_2009	[Adalimumab plus methotrexate for the treatment of rheumatoid arthritis: a multi-center randomized, double-blind, placebo-controlled clinical study.]	Zhonghua_Nei_Ke_Za_Zhi
Huang_2013	Infliximab reduces CD147, MMP-3, and MMP-9 expression in peripheral blood monocytes in patients with active rheumatoid arthritis	European_Journal_of_Pharmacology
Hunt_1997	The effects of daily intake of folic acid on the efficacy of methotrexate therapy in children with juvenile rheumatoid arthritis. A controlled study	Journal_of_Rheumatology
Iqbal_2015	Short Communication: Lack of association between MTHFR gene polymorphisms and response to methotrexate treatment in Pakistani patients with rheumatoid arthritis	Pak_J_Pharm_Sci
Ishaq_2011	Leflunomide or methotrexate? Comparison of clinical efficacy and safety in low socio-economic rheumatoid arthritis patients	Modern_Rheumatology
Ishiguro_2013	Concomitant iguratimod therapy in patients with active rheumatoid arthritis despite stable doses of methotrexate: A randomized, double-blind, placebo-controlled trial	Modern_Rheumatology
Islam_2000	Efficacy of sulphasalazine plus methotrexate in rheumatoid arthritis	Bangladesh_Medical_Research_Council_Bulletin
Islam_2013	Comparative efficacy of subcutaneous versus oral methotrexate in active rheumatoid arthritis	Mymensingh_Med_J
JaimesHernandes_2012	Efficacy of leflunomide 100 mg weekly compared to low dose methotrexate in patients with active rheumatoid arthritis. Double blind, randomized clinical trial. [Spanish]	Reumatologia_Clinica

Jarrett_2006	Preliminary evidence for a structural benefit of the new bisphosphonate zoledronic acid in early rheumatoid arthritis	Arthritis_Rheumatism
Jeurissen_1991	Influence of methotrexate and azathioprine on radiologic progression in rheumatoid arthritis. A randomized, double-blind study	Annals_of_internal_medicine
Jeurissen_1994	Methotrexate versus azathioprine in rheumatoid arthritis	Pharmacy_World_Science
Jiang_2010	[Effect of Bizhongxiao decoction on proteomics of peripheral blood mononuclear cells in patients with rheumatoid arthritis]. [Chinese]	Zhongguo_Zhong_Xi_Yi_Jie_He_Za_Zhi
Jie_2012	[Clinical study of Biqi Capsule combined with methotrexate for treatment of rheumatoid arthritis]. [Chinese]	Zhongguo_Zhong_Xi_Yi_Jie_He_Za_Zhi
Jing_2011	Relationship between RANKL/OPG system and type II collagen-induced arthritis in rats and the intervention of [⁹⁹Tc]-methylene diphosphonate. [Chinese]	Journal_of_China_Pharmaceutical_University
Johansen_1999	Serum YKL-40 concentrations in patients with rheumatoid arthritis: Relation to disease activity	Rheumatology
Jones_2010a	The AMBITION trial: Tocilizumab monotherapy for rheumatoid arthritis	Expert_Review_of_Clinical_Immunology
Jones_2010b	Comparison of tocilizumab monotherapy versus methotrexate monotherapy in patients with moderate to severe rheumatoid arthritis: The AMBITION study	Ann_Rheum_Dis
Jung_2015a	BRAin isolated from caesalpinia sappan L. inhibits rheumatoid arthritis activity in a type-II collagen induced arthritis mouse model	BMC_Complementary_Alternative_Medicine
Jung_2015b	Anti-inflammatory activity of sappanchalcone isolated from Caesalpinia sappan L. in a collagen-induced arthritis mouse model	Archives_of_Pharmacal_Research
Jurcovicova_2009	Methotrexate treatment ameliorated testicular suppression and anorexia related leptin reduction in rats with adjuvant arthritis	Rheumatology_International
Jurgens_2013	Increase of body mass index in a tight controlled methotrexate-based strategy with prednisone in early rheumatoid arthritis: side effect of the prednisone or better control of disease activity?	Arthritis_Care_Research
Jurgens_2014	The separate impact of tight control schemes and disease activity on quality of life in patients with early rheumatoid arthritis: Results from the CAMERA trials	Clinical_Experimental_Rheumatology
Kahn_2015	Effect of Withania somnifera (Ashwagandha) root extract on amelioration of oxidative stress and autoantibodies production in collagen-induced arthritic rats	Journal_of_Complementary_Integrative_Medicine
KaminskaTchorzewksa_2001	The evaluation of aggressive treatment (methotrexate + methylprednisolone) in patients with early rheumatoid arthritis	Reumatologia
Kang_2015	Development of the potent anti-rheumatoid arthritis compound derived from rosmarinic acid and the evaluation of the activity in collagen-induced arthritis mouse model	International_Journal_of_Pharmacology
Kavanaugh_2000	Chimeric anti-tumor necrosis factor-alpha monoclonal antibody treatment of patients with rheumatoid arthritis receiving methotrexate therapy	Journal_of_Rheumatology
Kavanaugh_2010	Is a 12-week trial sufficient to evaluate clinical responses to etanercept or MTX treatment in early RA?	Rheumatology

Kavanaugh_2013	Clinical, functional and radiographic consequences of achieving stable low disease activity and remission with adalimumab plus methotrexate or methotrexate alone in early rheumatoid arthritis: 26-week results from the randomised, controlled OPTIMA study	Ann_Rheum_Dis
Kawai_1997	Low-dose pulse methotrexate inhibits articular destruction of adjuvant arthritis in rats	Journal_of_Pharmacy_Pharmacology
Kay_2008	Golimumab in patients with active rheumatoid arthritis despite treatment with methotrexate: A randomized, double-blind, placebo-controlled, dose-ranging study	Arthritis_Rheumatism
Kekow_2010	Patient-reported outcomes improve with etanercept plus methotrexate in active early rheumatoid arthritis and the improvement is strongly associated with remission: The COMET trial	Ann_Rheum_Dis
Kerstens_1994	Antiperinuclear factor and disease activity in rheumatoid arthritis. Longitudinal evaluation during methotrexate and azathioprine therapy	J_Rheumatol
Kerstens_2000	Radiological and clinical results of longterm treatment of rheumatoid arthritis with methotrexate and azathioprine	Journal_of_Rheumatology
Kerwar_1989	Methotrexate in rheumatoid arthritis: studies with animal models	Advances_in_enzyme_regulation
Keystone_2004	Radiographic, Clinical, and Functional Outcomes of Treatment with Adalimumab (a Human Anti-Tumor Necrosis Factor Monoclonal Antibody) in Patients with Active Rheumatoid Arthritis Receiving Concomitant Methotrexate Therapy: A Randomized, Placebo-Controlled, 52-Week Trial	Arthritis_Rheumatism
Keystone_2005	B cells in rheumatoid arthritis: from hypothesis to the clinic	Rheumatology
Keystone_2008a	Certolizumab pegol plus methotrexate is significantly more effective than placebo plus methotrexate in active rheumatoid arthritis: Findings of a fifty-two-week, phase III, multicenter, randomized, double-blind, placebo-controlled, parallel-group study	Arthritis_Rheumatism
Keystone_2008b	Improvement in patient-reported outcomes in a rituximab trial in patients with severe rheumatoid arthritis refractory to anti-tumor necrosis factor therapy	Arthritis_Care_Research
Keystone_2009a	Golimumab, a human antibody to tumour necrosis factor alpha given by monthly subcutaneous injections, in active rheumatoid arthritis despite methotrexate therapy: The GO-FORWARD Study	Ann_Rheum_Dis
Keystone_2009b	Rituximab inhibits structural joint damage in patients with rheumatoid arthritis with an inadequate response to tumour necrosis factor inhibitor therapies	Ann_Rheum_Dis
Keystone_2010	Golimumab in patients with active rheumatoid arthritis despite methotrexate therapy: 52-week results of the GO-FORWARD study	Ann_Rheum_Dis
Keystone_2011a	Clinical consequences of delayed addition of adalimumab to methotrexate therapy over 5 years in patients with rheumatoid arthritis	Journal_of_Rheumatology
Keystone_2011b	Rapid improvement in the signs and symptoms of rheumatoid arthritis following certolizumab pegol treatment predicts better longterm outcomes: post-hoc analysis of a randomized controlled trial	J_Rheumatol
Keystone_2013	Golimumab in patients with active rheumatoid arthritis despite methotrexate therapy: Results through 2 years of the go-forward study extension	Journal_of_Rheumatology
Keystone_2014	Clinical, functional, and radiographic implications of time to treatment response in patients with early rheumatoid arthritis: a posthoc analysis of the PREMIER study	J_Rheumatol

Keystone_2016a	Longterm effect of delaying combination therapy with tumor necrosis factor inhibitor in patients with aggressive early rheumatoid arthritis: 10-year efficacy and safety of adalimumab from the randomized controlled PREMIER trial with open-label extension	Journal_of_Rheumatology
Keystone_2016b	Safety and efficacy of subcutaneous golimumab in patients with active rheumatoid arthritis despite methotrexate therapy: Final 5-year results of the GO-FORWARD trial	Journal_of_Rheumatology
Khan_2011a	Rituximab after methotrexate failure in rheumatoid arthritis: Evaluation of the SERENE trial	Expert_Opinion_on_Biological_Therapy
Khan_2011b	Clinical evaluation of herbal medicines for the treatment of rheumatoid arthritis	Pakistan_Journal_of_Nutrition
Kim_2000	Divergent effect of cyclosporine on Th1/Th2 type cytokines in patients with severe, refractory rheumatoid arthritis	Journal_of_Rheumatology
Kim_2007	A randomized, double-blind, placebo-controlled, phase III study of the human anti-tumor necrosis factor antibody adalimumab administered as subcutaneous injections in KORn rheumatoid arthritis patients treated with methotrexate	APLAR_Journal_of_Rheumatology
Kim_2012	Efficacy of concurrent administration of cilostazol and methotrexate in rheumatoid arthritis: Pharmacologic and clinical significance	Life_Sciences
Kim_2013	A clinical trial and extension study of infliximab in KORn patients with active rheumatoid arthritis despite methotrexate treatment	Journal_of_KORn_medical_science
Kimel_2008	Adalimumab plus methotrexate improved SF-36 scores and reduced the effect of rheumatoid arthritis (RA) on work activity for patients with early RA	Journal_of_Rheumatology
Kirkham_2014	Effects of golimumab, an anti-tumour necrosis factor-alpha human monoclonal antibody, on lipids and markers of inflammation	Ann_Rheum_Dis
Kita_2002	[The effect of low dose methotrexate treatment on bone mineral density in patients with rheumatoid arthritis]	Pol_Merkur_Lekarski
Klareskog_2004	Therapeutic effect of the combination of etanercept and methotrexate compared with each treatment alone in patients with rheumatoid arthritis: Double-blind randomised controlled trial	Lancet
Kochbati_2003	[Radiologic progression of rheumatoid arthritis lesions treated with methotrexate]	Tunis_Med
Koller_2009	Response of elderly patients with rheumatoid arthritis to methotrexate or TNF inhibitors compared with younger patients	Rheumatology
Kosinski_2002	Health-related quality of life in early rheumatoid arthritis: Impact of disease and treatment response	American_Journal_of_Managed_Care
Kosmaczewska_2014	Alterations in both the activatory and inhibitory potential of peripheral blood CD4+ T cells in rheumatoid arthritis patients correlate with disease progression	Pathol_Oncol_Res
Kosmaczewska_2015	Exogenous IL-2 controls the balance in Th1, Th17, and Treg cell distribution in patients with progressive rheumatoid arthritis treated with TNF-alpha inhibitors	Inflammation
Kovalenko_2001	Estimation of efficacy of basic therapy of rheumatoid arthritis on the basis of systemic enzyme therapy: Results of five-year monitoring	International_Journal_of_Immunotherapy
Kraan_2000	Modulation of inflammation and metalloproteinase expression in synovial tissue by leflunomide and methotrexate in patients with active rheumatoid arthritis. Findings in a prospective, randomized, double-blind, parallel-design clinical trial in thirty-nine patients at two centers	Arthritis_Rheumatism

Kraan_2004	Differential effects of leflunomide and methotrexate on cytokine production in rheumatoid arthritis	Ann_Rheum_Dis
Krausz_2012	A phase IIa, randomized, double-blind, placebo-controlled trial of apilimod mesylate, an interleukin-12/interleukin-23 inhibitor, in patients with rheumatoid arthritis	Arthritis_Rheumatism
Kremer_1995	Every-other-week methotrexate in patients with rheumatoid arthritis: A double-blind, placebo-controlled prospective study	Arthritis_Rheumatism
Kremer_2002	Concomitant leflunomide therapy in patients with active rheumatoid arthritis despite stable doses of methotrexate: A randomized, double-blind, placebo-controlled trial	Annals_of_Internal_Medicine
Kremer_2003	Treatment of Rheumatoid Arthritis by Selective Inhibition of T-Cell Activation with Fusion Protein CTLA4Ig	New_England_Journal_of_Medicine
Kremer_2005	Treatment of rheumatoid arthritis with the selective costimulation modulator abatacept: Twelve-month results of a phase IIb, double-blind, randomized, placebo-controlled trial	Arthritis_Rheumatism
Kremer_2010	Golimumab, a new human anti-tumor necrosis factor alpha antibody, administered intravenously in patients with active rheumatoid arthritis: Forty-eight-week efficacy and safety results of a phase III randomized, double-blind, placebo-controlled study	Arthritis_Rheumatism
Kremer_2011	Tocilizumab inhibits structural joint damage in rheumatoid arthritis patients with inadequate responses to methotrexate: Results from the double-blind treatment phase of a randomized placebo-controlled trial of tocilizumab safety and prevention of structural joint damage at one year	Arthritis_Rheumatism
Kremer_2012	A phase IIb dose-ranging study of the oral JAK inhibitor tofacitinib (CP-690,550) versus placebo in combination with background methotrexate in patients with active rheumatoid arthritis and an inadequate response to methotrexate alone	Arthritis_rheumatism
Krintel_2015	Prediction of treatment response to adalimumab: a double-blind placebo-controlled study of circulating microRNA in patients with early rheumatoid arthritis	Pharmacogenomics_J
Kroger_1999	The effect of tryptophan plus methionine, 5-azacytidine, and methotrexate on adjuvant arthritis of rat	General_Pharmacology
KudoTanaka_2015	Early therapeutic intervention with methotrexate prevents the development of rheumatoid arthritis in patients with recent-onset undifferentiated arthritis: A prospective cohort study	Modern_Rheumatology
Kuncirova_2014	N-feruloylserotonin in preventive combination therapy with methotrexate reduced inflammation in adjuvant arthritis	Fundamental_Clinical_Pharmacology
Kurasawa_2014	Addition of another disease-modifying anti-rheumatic drug to methotrexate reduces the flare rate within 2 years after infliximab discontinuation in patients with rheumatoid arthritis: An open, randomized, controlled trial	Modern_Rheumatology
Lacki_1995	Circulating interleukin 10 and interleukin-6 serum s in rheumatoid arthritis patients treated with methotrexate or gold salts: preliminary report	Inflamm_Res
Lacki_1996	The influence of methotrexate on serum of agalactosyl IgG in rheumatoid arthritis patients	Central-European_Journal_of_Immunology
Lafforgue_1995	Lack of correlation between pharmacokinetics and efficacy of low dose methotrexate in patients with rheumatoid arthritis	Journal_of_Rheumatology

Lambert_2004	Dose Escalation of Parenteral Methotrexate in Active Rheumatoid Arthritis That Has Been Unresponsive to Conventional Doses of Methotrexate: A Randomized, Controlled Trial	Arthritis_Rheumatism
Lan_2004	A comparative study of etanercept plus methotrexate and methotrexate alone in Taiwanese patients with active rheumatoid arthritis: A 12-week, double-blind, randomized, placebo-controlled study	Journal_of_the_Formosan_Medical_Association
Landewe_2006	Disconnect between inflammation and joint destruction after treatment with etanercept plus methotrexate: Results from the trial of etanercept and methotrexate with radiographic and patient outcomes	Arthritis_Rheumatism
Landewe_2015	Existing joint erosions increase the risk of joint space narrowing independently of clinical synovitis in patients with early rheumatoid arthritis	Arthritis_Res_Ther
Lange_2005	Methotrexate ameliorates T cell dependent autoimmune arthritis and encephalomyelitis but not antibody induced or fibroblast induced arthritis	Ann_Rheum_Dis
Laurberg_2009	Plasma adiponectin in patients with active, early, and chronic rheumatoid arthritis who are steroid- and disease-modifying antirheumatic drug-naive compared with patients with osteoarthritis and controls	J_Rheumatol
Laustsen_2014	Soluble OX40L is associated with presence of autoantibodies in early rheumatoid arthritis	Arthritis_Res_Ther
Lee_2009	The efficacy and mechanism action of rvcsd, a new herbal agent, on immune suppression and cartilage protection in a mouse model of rheumatoid arthritis	Journal_of_Pharmacological_Sciences
Lee_2012	Alleviation of rheumatoid arthritis by cell-transducible methotrexate upon transcutaneous delivery	Biomaterials
Lee_2014a	Tofacitinib versus methotrexate in rheumatoid arthritis	New_England_Journal_of_Medicine
Lee_2014b	Prediction of antiarthritic drug efficacies by monitoring active matrix metalloproteinase-3 (MMP-3) s in collagen-induced arthritic mice using the MMP-3 probe	Molecular_Pharmaceutics
LeGoff_2009	A combination of methotrexate and zoledronic acid prevents bone erosions and systemic bone mass loss in collagen induced arthritis	Arthritis_Res_Ther
Lehman_2005	A 48-week, randomized, double-blind, double-observer, placebo-controlled multicenter trial of combination methotrexate and intramuscular gold therapy in rheumatoid arthritis: Results of the METGO study	Arthritis_Rheumatism
Lerndal_2000	A clinical study of CPH 82 vs methotrexate in early rheumatoid arthritis	Rheumatology
Levitsky_2015a	Predicted vs. observed radiographic progression in early rheumatoid arthritis (POPeRA): Results from a randomized trial	Scandinavian_Journal_of_Rheumatology
Levitsky_2015b	Serum survivin predicts responses to treatment in active rheumatoid arthritis: A post hoc analysis from the SWEFOT trial	BMC_Medicine
Li_2008	Decreased external home help use with improved clinical status in rheumatoid arthritis: An exploratory analysis of the Abatacept in Inadequate Responders to Methotrexate (AIM) trial	Clinical_Therapeutics
Li_2015	Efficacy and safety results from a Phase 3, randomized, placebo-controlled trial of subcutaneous golimumab in Chinese patients with active rheumatoid arthritis despite methotrexate therapy	Int_J_Rheum_Dis

Liang_2000	[Clinical investigation of effects of bizhongxiao decoction (BZX) on rheumatoid arthritis on active phase]	Hunan_Yi_Ke_Da_Xue_Xue_Bao
Liang_2002	Effect of bizhongxiao decoction on the expression of VEGF in the synovial membrane of C II-induced rheumatoid arthritis in rats. [Chinese]	Bulletin_of_Hunan_Medical_University
Liang_2004	Effect of bizhongxiao decoction on the expression of vascular endothelial growth factor in the synovial membrane and symptom of rheumatoid arthritis in rats	Chinese_Journal_of_Clinical_Rehabilitation
Liang_2009	Intra-articular treatment of inflammatory arthritis with microsphere formulations of methotrexate: Pharmacokinetics and efficacy determination in antigen-induced arthritic rabbits	Inflammation_Research
Lin_2009	Curative effect of glucosamine hydrochloride administered alone or combined with methotrexate on rat model of adjuvant arthritis. [Chinese]	Chinese_Journal_of_Biologicals
Lin_2011	[Multi-center clinical study on therapeutic effect of kunxian capsule on rheumatoid arthritis]. [Chinese]	Zhongguo_Zhong_Xi_Yi_Jie_He_Za_Zhi
Lin_2014	Anti-arthritic activity of Xanthium strumarium L. Extract on complete Freund's adjuvant induced arthritis in rats	Journal_of_Ethnopharmacology
Lina_2011	Combined treatment of etanercept and MTX reverses Th1/Th2, Th17/Treg imbalance in patients with rheumatoid arthritis	J_Clin_Immunol
Liu_2006	Effects of traditional Chinese medicine for invigorating spleen to resolve dampness and dredging collaterals on patients with rheumatoid arthritis and anemia. [Chinese]	Journal_of_Chinese_Integrative_Medicine
Liu_2008	Effects of Tongbi Mixture 2 on expressions of CD28 and CD152 and content of tumor necrosis factor-alpha in peripheral blood in rats with collagen-induced arthritis. [Chinese]	Journal_of_Chinese_Integrative_Medicine
Liu_2013	Pharmacokinetics, pharmacodynamics and toxicities of methotrexate in healthy and collagen-induced arthritic rats	Biopharmaceutics_Drug_Disposition
LopezMendez_1993	Radiographic assessment of disease progression in rheumatoid arthritis patients enrolled in the cooperative systematic studies of the rheumatic diseases program randomized clinical trial of methotrexate, auranofin, or a combination of the two	Arthritis_Rheumatism
Lu_2009	Multicenter, randomized, double-blind, controlled trial of treatment of active rheumatoid arthritis with T-614 compared with methotrexate	Arthritis_Care_Research
Luis_1999	Comparison of two schedules for administering oral low-dose methotrexate (weekly versus every-other-week) in patients with rheumatoid arthritis in remission: A twenty-four-week, single-blind, randomized study	Arthritis_Rheumatism
Luo_2013	A novel disease-modifying antirheumatic drug, iguratimod, ameliorates murine arthritis by blocking IL-17 signaling, distinct from methotrexate and leflunomide	Journal_of_Immunology
Lv_2015	Comparison of Tripterygium wilfordii Hook F with methotrexate in the treatment of active rheumatoid arthritis (TRIFRA): A randomised, controlled clinical trial	Ann_Rheum_Dis
Magari_2004	Comparison of anti-arthritic properties of leflunomide with methotrexate and FK506: Effect on T cell activation-induced inflammatory cytokine production in vitro and rat adjuvant-induced arthritis	Inflammation_Research
MaI_2014	Clinical and serological predictors of remission in rheumatoid arthritis are dependent on treatment regimen	Journal_of_Rheumatology

Maillefert_2003	Long term structural effects of combination therapy in patients with early rheumatoid arthritis: Five year follow up of a prospective double blind controlled study	Ann_Rheum_Dis
Maini_1998	Therapeutic efficacy of multiple intravenous infusions of anti-tumor necrosis factor monoclonal antibody combined with low-dose weekly methotrexate in rheumatoid arthritis	Arthritis_Rheumatism
Maini_1999	Infliximab (chimeric anti-tumour necrosis factor alpha monoclonal antibody) versus placebo in rheumatoid arthritis patients receiving concomitant methotrexate: A randomised phase III trial	Lancet
Maini_2006	Double-blind randomized controlled clinical trial of the interleukin-6 receptor antagonist, tocilizumab, in European patients with rheumatoid arthritis who had an incomplete response to methotrexate	Arthritis_Rheumatism
Majumdar_2012	Methotrexate (MTX)-cIBR conjugate for targeting MTX to leukocytes: Conjugate stability and in vivo efficacy in suppressing rheumatoid arthritis	Journal_of_Pharmaceutical_Sciences
Makrygiannakis_2012	Local administration of glucocorticoids decreases synovial citrullination in rheumatoid arthritis	Arthritis_Res_Ther
Marchesoni_2003	Radiographic progression in early rheumatoid arthritis: A 12-month randomized controlled study comparing the combination of cyclosporin and methotrexate with methotrexate alone	Rheumatology
Marchesoni_2005	Cyclosporine in addition to infliximab and methotrexate in refractory rheumatoid arthritis [7]	Clinical_Experimental_Rheumatology
Masatoshi Hayashi_2013	Golimumab reduces disease activity of rheumatoid arthritis for 1 year and strongly inhibits radiographic progression in Japanese patients: Partial but detailed results of the GO-FORTH and GO-MONO studies	Clinical_Rheumatology
Matsuno_2007	Requirement of methotrexate in combination with anti-tumor necrosis factor-alpha therapy for adequate suppression of osteoclastogenesis in rheumatoid arthritis	Journal_of_Rheumatology
Mazurov_2014	The quality of life in patients with rheumatoid arthritis treated with rituximab. [Russian]	Klinicheskaia_meditsina
McKendry_1990	Azathioprine and methotrexate as combination chemotherapy in rheumatoid arthritis	Journal_of_Rheumatology
Meade_2013	A preliminary investigation of cognitive function in rheumatoid arthritis patients on long-term methotrexate treatment	Journal_of_health_psychology
Mease_2008	Improved health-related quality of life for patients with active rheumatoid arthritis receiving rituximab - Results of the dose-ranging assessment: International clinical evaluation of rituximab in rheumatoid arthritis (DANCER) trial	Journal_of_Rheumatology
Mease_2012	A phase II, double-blind, randomised, placebo-controlled study of BMS945429 (ALD518) in patients with rheumatoid arthritis with an inadequate response to methotrexate	Ann_Rheum_Dis
Mehta_2012	Anti-arthritis activity of roots of Hemidesmus indicus R.Br. (Anantmul) in rats	Asian_Pacific_Journal_of_Tropical_Medicine
Mello_2013	Intra-articular methotrexate associated to lipid nanoemulsions: Anti-inflammatory effect upon antigen-induced arthritis	International_Journal_of_Nanomedicine
Menninger_1996	Radiographic healing phenomena in rheumatoid arthritis treated with methotrexate or goldsodiumthiomalate. [German]	Zeitschrift_fur_Rheumatologie
Menninger_1998	A 36 month comparative trial of methotrexate and gold sodium thiomalate in the treatment of early active and erosive rheumatoid arthritis	British_Journal_of_Rheumatology

Michaels_1996	Serum phospholipase A<inf>2</inf> activity in patients with rheumatoid arthritis before and after treatment with methotrexate, auranofin, or combination of the two	Journal_of_Rheumatology
Mielants_1991	The efficacy and toxicity of a constant low dose of methotrexate as a treatment for intractable rheumatoid arthritis: an open prospective study	J_Rheumatol
Mihara_1996	In vitro and in vivo biological activities of a novel nonpolyglutamable anti-folate, MX-68	Immunopharmacology
Mikhael_2013	Effect of rosuvastatin as adjuvant therapy to methotrexate on hematological parameters in patients with moderately-highly active rheumatoid arthritis	Journal_of_Experimental_Integrative_Medicine
Mirshafiey_2006	Design of a new line in treatment of experimental rheumatoid arthritis by artesunate	Immunopharmacology_Immunotoxicology
Montecucco_2012	Low-dose oral prednisone improves clinical and ultrasonographic remission rates in early rheumatoid arthritis: Results of a 12-month open-label randomised study	Arthritis_Res_Ther
Morassut_1989	Gold sodium thiomalate compared to low dose methotrexate in the treatment of rheumatoid arthritis--a randomized, double blind 26-week trial	J_Rheumatol
Moreland_1995	Double-blind, placebo-controlled multicenter trial using chimeric monoclonal anti-CD4 antibody, cM-T412, in rheumatoid arthritis patients receiving concomitant methotrexate	Arthritis_Rheumatism
Moreland_2006	Effect of etanercept on fatigue in patients with recent or established rheumatoid arthritis	Arthritis_Care_Research
Morgan_1994	Supplementation with folic acid during methotrexate therapy for rheumatoid arthritis. A double-blind, placebo-controlled trial	Annals_of_Internal_Medicine
Morgan_2001	MTX affects inflammation and tissue destruction differently in the rat AA model	Journal_of_Rheumatology
Morgan_2004	Effect of methotrexate therapy on bone mineral density and body composition in rat adjuvant arthritis	Journal_of_Rheumatology
Moroldo_1998	Estimates of the discriminant ability of definitions of improvement for juvenile rheumatoid arthritis	Journal_of_Rheumatology
Murav_2014	[Evaluation of methotrexate effect on the acute-phase response in rheumatoid arthritis after 12-week treatment]. [Russian]	Klinicheskaia_meditsina
Murayama_1992	[The treatment of rheumatoid arthritis with low dose pulse methotrexate--comparative study with other disease modifying antirheumatic drugs]	Ryumachi
Nagate_2007	Tranilast suppresses the disease development of the adjuvant- and streptococcal cell wall-induced arthritis in rats	Journal_of_Pharmacological_Sciences
Nagate_2009	Therapeutic and preventive effects of methotrexate on zymosan-induced arthritis in SKG mice	Journal_of_Veterinary_Medical_Science
Nakazawa_2001	Methotrexate inhibits rheumatoid synovitis by inducing apoptosis	Journal_of_Rheumatology
Nam_2014	A randomised controlled trial of etanercept and methotrexate to induce remission in early inflammatory arthritis: The EMPIRE trial	Ann_Rheum_Dis
NavarroMillan_2013	Changes in lipoproteins associated with methotrexate or combination therapy in early rheumatoid arthritis: results from the treatment of early rheumatoid arthritis trial	Arthritis_Rheumatism
Nesher_1997	Effect of treatment with methotrexate, hydroxychloroquine, and prednisone on lymphocyte polyamine s in rheumatoid arthritis: correlation with the clinical response and rheumatoid factor synthesis	Clin_Exp_Rheumatol

Neurath_1999	Methotrexate specifically modulates cytokine production by T cells and macrophages in murine collagen-induced arthritis (CIA): A mechanism for methotrexate-mediated immunosuppression	Clinical_Experimental_Immunology
Niu_2011	Regulatory immune responses induced by IL-1 receptor antagonist in rheumatoid arthritis	Mol_Immunol
Nowak_2010	High-dose methotrexate ameliorates collagen-induced arthritis but does not inhibit the release of proinflammatory cytokines by peritoneal macrophages in mice	Central-European_Journal_of_Immunology
Ochaion_2006	Methotrexate enhances the anti-inflammatory effect of CF101 via up-regulation of the A3 adenosine receptor expression	Arthritis_Res_Ther
Odell_1996	Efficacy of triple DMARD therapy in patients with RA with suboptimal response to methotrexate	Journal_of_Rheumatology
Odell_2006	Treatment of early seropositive rheumatoid arthritis: Doxycycline plus methotrexate versus methotrexate alone	Arthritis_Rheumatism
Ogrendik_2007	Levofloxacin treatment in patients with rheumatoid arthritis receiving methotrexate	Southern_Medical_Journal
Oliunin_1996	Combined immunomodulating therapy in rheumatoid arthritis. [Russian]	Terapevticheskii_arkhiv
Olsen_1991	IgM-rheumatoid factor and responses to second-line drugs in rheumatoid arthritis	Agents_Actions
Omata_1997	Z-100, extracted from Mycobacterium tuberculosis strain Aoyama B, inhibits the development of collagen-induced arthritis in mice	Biological_Pharmaceutical_Bulletin
Ostergaard_2011	Significant improvement in synovitis, osteitis, and bone erosion following golimumab and methotrexate combination therapy as compared with methotrexate alone: A magnetic resonance imaging study of 318 methotrexate-naive rheumatoid arthritis patients	Arthritis_Rheumatism
Pandya_2002	Methotrexate twice weekly vs once weekly in rheumatoid arthritis: A pilot double-blind, controlled study	Rheumatology_International
Park_2015	DC-Based Immunotherapy Combined with Low-Dose Methotrexate Effective in the Treatment of Advanced CIA in Mice	Journal_of_Immunology_Research
Park_2016	Treatment of collagen-induced arthritis using immune modulatory properties of human mesenchymal stem cells	Cell_Transplant
Parker_2004	Subtherapeutic Dosing of Methotrexate in Rheumatoid Arthritis Trials [2]	Journal_of_the_American_Osteopathic_Association
Patel_2009a	Methotrexate versus combination methotrexate and etanercept for rheumatoid arthritis	Current_Rheumatology_Reports
Patel_2009b	Tocilizumab versus methotrexate in moderate to severe rheumatoid arthritis	Current_Rheumatology_Reports
Patten_2004	Characterization of pristane-induced arthritis, a murine model of chronic disease: Response to antirheumatic agents, expression of joint cytokines, and immunopathology	Arthritis_Rheumatism
Paulus_1990	Analysis of improvement in individual rheumatoid arthritis patients treated with disease-modifying antirheumatic drugs, based on the findings in patients treated with placebo. The Cooperative Systematic Studies of Rheumatic Diseases Group	Arthritis_Rheumatism
Pavelka_2014	Etanercept in moderate rheumatoid arthritis: PRESERVE study results from central/eastern Latin America and Asia	International_Journal_of_Clinical_Rheumatology
Perkins_1998	Reduction of NOS2 overexpression in rheumatoid arthritis patients treated with anti-tumor necrosis factor a monoclonal antibody (cA2)	Arthritis_Rheumatism
Peterfy_2013	Monitoring cartilage loss in the hands and wrists in rheumatoid arthritis with magnetic resonance imaging in a multi-center clinical trial: IMPRESS (NCT00425932)	Arthritis_Res_Ther

Peterfy_2016	Sustained improvements in MRI outcomes with abatacept following the withdrawal of all treatments in patients with early, progressive rheumatoid arthritis	Ann_Rheum_Dis
Pigott_2014	1-Methyl-tryptophan synergizes with methotrexate to alleviate arthritis in a mouse model of arthritis	Autoimmunity
Pincus_2011	RAPID3 (Routine Assessment of Patient Index Data 3) severity categories and response criteria: Similar results to DAS28 (Disease Activity Score) and CDAI (Clinical Disease Activity Index) in the RAPID 1 (Rheumatoid Arthritis Prevention of Structural Damage) clinical trial of certolizumab pegol	Arthritis_Care_Research
Pinheiro_1993	A short-term randomized controlled study with methotrexate in rheumatoid arthritis. [Portuguese]	Revista_da_Associacao_Medica_Brasileira_(1992)
Ponchel_2014	An immunological biomarker to predict MTX response in early RA	Ann_Rheum_Dis
Popovic_1998	Comparative study of the clinical efficacy of four DMARDs (Leflunomide, methotrexate, cyclosporine, and levamisole) in patients with rheumatoid arthritis	Transplantation_Proceedings
Quinn_2005	Very early treatment with infliximab in addition to methotrexate in early, poor-prognosis rheumatoid arthritis reduces magnetic resonance imaging evidence of synovitis and damage, with sustained benefit after infliximab withdrawal: Results from a twelve-month randomized, double-blind, placebo-controlled trial	Arthritis_Rheumatism
Rau_1991	A double-blind comparison of parenteral methotrexate and parenteral gold in the treatment of early erosive rheumatoid arthritis: an interim report on 102 patients after 12 months	Semin_Arthritis_Rheum
Rau_1997a	Long-term treatment of destructive rheumatoid arthritis with methotrexate	J_Rheumatol
Rau_1997b	Comparison of intramuscular methotrexate and gold sodium thiomalate in the treatment of early erosive rheumatoid arthritis: 12 month data of a double-blind parallel study of 174 patients	British_Journal_of_Rheumatology
Rau_1998	Progression in early erosive rheumatoid arthritis: 12 month results from a randomized controlled trial comparing methotrexate and gold sodium thiomalate	British_Journal_of_Rheumatology
Rau_2002	Radiographic outcome after three years of patients with early erosive rheumatoid arthritis treated with intramuscular methotrexate or parenteral gold. Extension of a one-year double-blind study in 174 patients	Rheumatology
Rau_2004	Rapid alleviation of signs and symptoms of rheumatoid arthritis with intravenous or subcutaneous administration of adalimumab in combination with methotrexate	Scandinavian_Journal_of_Rheumatology
Reece_2002	Comparative assessment of leflunomide and methotrexate for the treatment of rheumatoid arthritis, by dynamic enhanced magnetic resonance imaging	Arthritis_Rheumatism
Refaat_2013	Evaluation of the effect of losartan and methotrexate combined therapy in adjuvant-induced arthritis in rats	European_Journal_of_Pharmacology
RellBakalarska_1998	Assessment of the results of combined treatment with cyclophosphamide and prednisone or methotrexate and prednisone in patients with rheumatoid arthritis with concomitant vasculitis. [Polish]	Reumatologia
Revu_2013	Synovial membrane immunohistology in early-untreated rheumatoid arthritis reveals high expression of catabolic bone markers that is modulated by methotrexate	Arthritis_Res_Ther

Rezaei_2012	In early rheumatoid arthritis, patients with a good initial response to methotrexate have excellent 2-year clinical outcomes, but radiological progression is not fully prevented: data from the methotrexate responders population in the SWEFOT trial	Ann_Rheum_Dis
Rigby_2012	Safety and efficacy of ocrelizumab in patients with rheumatoid arthritis and an inadequate response to methotrexate: Results of a forty-eight-week randomized, double-blind, placebo-controlled, parallel-group phase III trial	Arthritis_Rheumatism
Riksen_2006	Methotrexate modulates the kinetics of adenosine in humans in vivo	Ann_Rheum_Dis
RomanBlas_2010	Efficacy and safety of a selective estrogen receptor beta agonist, ERB-041, in patients with rheumatoid arthritis: a 12-week, randomized, placebo-controlled, phase II study	Arthritis_Care_Research
Ronda_2015	Newly identified antiatherosclerotic activity of methotrexate and adalimumab: complementary effects on lipoprotein function and macrophage cholesterol metabolism	Arthritis_Rheumatol
Rovensky_2002	Treatment of experimental adjuvant arthritis with the combination of methotrexate and lyophilized Enterococcus faecium enriched with organic selenium	Folia_microbiologica
Rovensky_2008	Effects of purified micronized flavonoid fraction (detralex) on prophylactic treatment of adjuvant arthritis with methotrexate in rats	ISR_Medical_Association_Journal
Rovensky_2009a	Treatment of rat adjuvant arthritis with flavonoid (Detralex), methotrexate, and their combination	Contemporary_Challenges_in_Autoimmunity
Rovensky_2009b	Treatment of adjuvant-induced arthritis with the combination of methotrexate and probiotic bacteria escherichia coli o83 (colinfant)	Folia_Microbiologica
rovensky_2011	The effects of synthetic immunostimulator polyoxidonium on methotrexate treatment in rats with adjuvant arthritis. [Polish, English]	Reumatologia
rovensky_2013	The effects of beta-glucan isolated from Pleurotus ostreatus on the development of arthritis and methotrexate treatment in rats with adjuvant arthritis	Reumatologia
Roy_2012	A novel combination of methotrexate and epigallocatechin attenuates the overexpression of pro-inflammatory cartilage cytokines and modulates antioxidant status in adjuvant arthritic rats	Inflammation
Rudwaleit_2000	Response to methotrexate in early rheumatoid arthritis is associated with a decrease of T cell derived tumour necrosis factor alpha, increase of interleukin 10, and predicted by the initial concentration of interleukin 4	Ann_Rheum_Dis
Ruperto_2007	A randomized, placebo-controlled trial of infliximab plus methotrexate for the treatment of polyarticular-course juvenile rheumatoid arthritis	Arthritis_Rheumatism
Saadat_2005	Effect of pyrimethamine in experimental rheumatoid arthritis	Medical_Science_Monitor
Saevarsdottir_2010	Predictors of response to methotrexate in early DMARD naive rheumatoid arthritis: results from the initial open-label phase of the SWEFOT trial	Ann_Rheum_Dis
Sakyo_1996	Inhibitory effect of methotrexate on neutrophil infiltration in carrageenan inflamed air pouch model of rats. [Japanese]	Pharmacometrics
Salaffi_1995a	Serum soluble interleukin-2 receptor s in rheumatoid arthritis: Effect of methotrexate, sulphasalazine and hydroxychloroquine therapy	Clinical_Rheumatology

Salaffi_1995b	A prospective study of the long-term efficacy and toxicity of low-dose methotrexate in rheumatoid arthritis	Clinical_Experimental_Rheumatology
Salaffi_1996	Three-year prospective study on methotrexate in rheumatoid arthritis. Analysis of radiological progression and comparison with a retrospective study [2]	Clinical_Experimental_Rheumatology
Salaza_2014	Polymorphisms in genes involved in the mechanism of action of methotrexate: are they associated with outcome in rheumatoid arthritis patients?	Pharmacogenomics
Salesi_2012	Efficacy of Vitamin D in patients with active rheumatoid arthritis receiving methotrexate therapy	Rheumatology_International
Salesi_2013	The role of bromocriptine in the treatment of patients with active rheumatoid arthritis	International_Journal_of_Rheumatic_Diseases
Sany_1991	Treatment of rheumatoid arthritis with methotrexate: a prospective open longterm study of 191 cases	J_Rheumatol
Sasakawa_2005	FK506 ameliorates spontaneous locomotor activity in collagen-induced arthritis: Implication of distinct effect from suppression of inflammation	International_Immunopharmacology
Schiff_1999	Leflunomide versus methotrexate: A comparison of the European and American experience	Scandinavian_Journal_of_Rheumatology
Schiff_2008	Efficacy and safety of abatacept or infliximab vs placebo in ATTEST: A phase III, multi-centre, randomised, double-blind, placebo-controlled study in patients with rheumatoid arthritis and an inadequate response to methotrexate	Ann_Rheum_Dis
Schipper_2009	Methotrexate therapy in rheumatoid arthritis after failure to sulphasalazine: to switch or to add?	Rheumatology
Schnabel_1994	Side effects and efficacy of 15 mg and 25 mg methotrexate per week in rheumatoid arthritis. [German]	Zeitschrift_fur_Rheumatologie
Schwager_2009	Preclinical characterization of DEKAVIL (F8-IL10), a novel clinical-stage immunocytokine which inhibits the progression of collagen-induced arthritis	Arthritis_Res_Ther
Scott_2016	A randomised trial evaluating anakinra in early active rheumatoid arthritis	Clin_Exp_Rheumatol
Seegobin_2014	ACPA-positive and ACPA-negative rheumatoid arthritis differ in their requirements for combination DMARDs and corticosteroids: Secondary analysis of a randomized controlled trial	Arthritis_Res_Ther
Segal_1989	Short term effects of low dose methotrexate on the acute phase reaction in patients with rheumatoid arthritis	J_Rheumatol
Segawa_1999	Age-depending effects of methotrexate treatment on systemic bone turnover in experimental adjuvant arthritis	Arzneimittel-Forschung_Drug_Research
Seideman_1993a	Methotrexate--the relationship between dose and clinical effect	Br_J_Rheumatol
Seideman_1993b	Better effect of methotrexate on C-reactive protein during daily compared to weekly treatment in rheumatoid arthritis	Clin_Rheumatol
Seitz_2000	Enhanced production of tissue inhibitor of metalloproteinases by peripheral blood mononuclear cells of rheumatoid arthritis patients responding to methotrexate treatment	Rheumatology
Seitz_2003	Pretreatment cytokine profiles of peripheral blood mononuclear cells and serum from patients with rheumatoid arthritis in different american college of rheumatology response groups to methotrexate	J_Rheumatol
Sergiets_2009	Combined basic therapy of rheumatoid arthritis with methotrexate and plaquenil. [Russian]	Terapevticheskii_arkhiv

Shanin_2011	Effect of peroxisome proliferator-activated receptor gamma agonist (pioglitazone) and methotrexate on disease activity in rheumatoid arthritis (experimental and clinical study)	Clinical_Medicine_Insights:_Arthritis_Musculoskeletal_Disorders
Sharp_2000	Treatment with leflunomide slows radiographic progression of rheumatoid arthritis: Results from three randomized controlled trials of leflunomide in patients with active rheumatoid arthritis	Arthritis_Rheumatism
Shen_2010	Infliximab reduces the frequency of interleukin 17-producing cells and the amounts of interleukin 17 in patients with rheumatoid arthritis	J_Investig_Med
Shikata_1996a	Evaluation of antirheumatic action of methotrexate (MTX) (2) effects on collagen induced arthritis in rats of oral pulse MTX. [Japanese]	Pharmacometrics
Shikata_1996b	Evaluation of antirheumatic action of methotrexate (MTX) (1) effects of MTX and 7-hydroxymethotrexate on collagen-induced arthritis and studies on the analgesic, anti-inflammatory and antipyretic actions in rats. [Japanese]	Pharmacometrics
Shilkina_2011	[Methotrexate-induced changes in the concentration of antibodies to mutaded citrullinated vimentin in blood serum of patients with rheumatoid arthritis]	Ter_Arkh
Shiroky_1993	Low-dose methotrexate with leucovorin (folinic acid) in the management of rheumatoid arthritis: Results of a multicenter randomized, double-blind, placebo-controlled trial	Arthritis_Rheumatism
Sigidin_1994	Comparative study of cyclosporin A in systemic rheumatoid arthritis	International_Journal_of_Immunotherapy
Silva_2006	Application of surface roughness analysis on micro-computed tomographic images of bone erosion: examples using a rodent model of rheumatoid arthritis	Molecular_imaging_:_official_journal_of_the_Society_for_Molecular_Imaging
Silverman_2005	Leflunomide or methotrexate for juvenile rheumatoid arthritis	New_England_Journal_of_Medicine
Simjee_2007	Quantitative gait analysis as a method to assess mechanical hyperalgesia modulated by disease-modifying antirheumatoid drugs in the adjuvant-induced arthritic rat	Arthritis_Res_Ther
Singh_2000	The open randomised trial of cyclosporine vs methotrexate in refractory rheumatoid arthritis	Journal_of_Internal_Medicine_of_IND
Smolen_2000	Efficacy and safety of leflunomide in active rheumatoid arthritis	Rheumatology
Smolen_2005	Evidence of radiographic benefit of treatment with infliximab plus methotrexate in rheumatoid arthritis patients who had no clinical improvement: a detailed subanalysis of data from the anti-tumor necrosis factor trial in rheumatoid arthritis with concomitant therapy study	Arthritis_Rheumatism
Smolen_2006a	Predictors of joint damage in patients with early rheumatoid arthritis treated with high-dose methotrexate with or without concomitant infliximab: Results from the ASPIRE trial	Arthritis_Rheumatism
Smolen_2006b	Infliximab treatment maintains employability in patients with early rheumatoid arthritis	Arthritis_Rheumatism
Smolen_2008a	Effect of interleukin-6 receptor inhibition with tocilizumab in patients with rheumatoid arthritis (OPTION study): a double-blind, placebo-controlled, randomised trial	Lancet
Smolen_2008b	Radiographic changes in rheumatoid arthritis patients attaining different disease activity states with methotrexate monotherapy and infliximab plus methotrexate: the impacts of remission and tumour necrosis factor blockade	Ann_Rheum_Dis
Smolen_2008c	Progression of radiographic joint damage in rheumatoid arthritis: independence of erosions and joint space narrowing	Ann_Rheum_Dis
Smolen_2009	Efficacy and safety of certolizumab pegol plus methotrexate in active rheumatoid arthritis: The RAPID 2 study. A randomised controlled trial	Ann_Rheum_Dis

Smolen_2012	Tocilizumab inhibits progression of joint damage in rheumatoid arthritis irrespective of its anti-inflammatory effects: Disassociation of the link between inflammation and destruction	Ann_Rheum_Dis
Smolen_2013	Maintenance, reduction, or withdrawal of etanercept after treatment with etanercept and methotrexate in patients with moderate rheumatoid arthritis (PRESERVE): A randomised controlled trial	Lancet
Smolen_2014	Adjustment of therapy in rheumatoid arthritis on the basis of achievement of stable low disease activity with adalimumab plus methotrexate or methotrexate alone: The randomised controlled OPTIMA trial	Lancet
Smolen_2015	Efficacy and safety of tabalumab, an anti-B-cellactivating factor monoclonal antibody, in patients with rheumatoid arthritis who had an inadequate response to methotrexate therapy: Results from a phase III multicentre, randomised, double-blind study	Ann_Rheum_Dis
Song_2007	Effect of artesunate on the expression of Fas/FasL and Bcl-2/Bax in synoviocytes of rats with adjuvant arthritis	Journal_of_Clinical_Rehabilitative_Tissue_Engineering_Research
Soubrier_2009	Evaluation of two strategies (initial methotrexate monotherapy vs its combination with adalimumab) in management of early active rheumatoid arthritis: data from the GUEPARD trial	Rheumatology
Spadaro_1993	One year treatment with low dose methotrexate in rheumatoid arthritis: effect on class specific rheumatoid factors	Clin_Rheumatol
Sreekanth_2000	Doxycycline in the treatment of rheumatoid arthritis--a pilot study	The_Journal_of_the_Association_of_Physicians_of_IND
StClair_2004	Combination of infliximab and methotrexate therapy for early rheumatoid arthritis: A randomized, controlled trial	Arthritis_Rheumatism
Stock_2012	Efficacy and safety of CE-224,535, an antagonist of P2X<inf>7</inf> receptor, in treatment of patients with rheumatoid arthritis inadequately controlled by methotrexate	Journal_of_Rheumatology
Stohl_2011	Safety and efficacy of ocrelizumab in combination with methotrexate in MTX-naive subjects with rheumatoid arthritis: The phase III FILM trial	Ann_Rheum_Dis
Stojanovic_2011	[Association of TNF-alpha polymorphism (-308 A/G) with high activity of rheumatoid arthritis and therapy response to Etanercept]	Srp_Arh_Celok_Lek
Strand_1999a	Treatment of active rheumatoid arthritis with leflunomide compared with placebo and methotrexate	Archives_of_Internal_Medicine
Strand_1999b	Function and health-related quality of life: Results from a randomized controlled trial of leflunomide versus methotrexate or placebo in patients with active rheumatoid arthritis	Arthritis_Rheumatism
Strand_2005	Physical function and health related quality of life: analysis of 2-year data from randomized, controlled studies of leflunomide, sulfasalazine, or methotrexate in patients with active rheumatoid arthritis	J_Rheumatol
Strand_2006	Sustained benefit in rheumatoid arthritis following one course of rituximab: Improvements in physical function over 2 years	Rheumatology

Strand_2009	Rapid and sustained improvements in health-related quality of life, fatigue, and other patient-reported outcomes in rheumatoid arthritis patients treated with certolizumab pegol plus methotrexate over 1 year: Results from the RAPID 1 randomized controlled trial	Arthritis_Res_Ther
Strand_2015	Tofacitinib with methotrexate in third-line treatment of patients with active rheumatoid arthritis: Patient-reported outcomes from a phase III trial	Arthritis_Care_Research
Straub_1997	Decrease of interleukin 6 during the first 12 months is a prognostic marker for clinical outcome during 36 months treatment with disease-modifying anti-rheumatic drugs	British_Journal_of_Rheumatology
SuaresAlmazor_1990	Parenteral methotrexate or gold for rheumatoid arthritis: a follow up	Clin_Exp_Rheumatol
SuarezAlmazor_1988	A randomized controlled trial of parenteral methotrexate compared with sodium aurothiomalate (myochrysine) in the treatment of rheumatoid arthritis	Journal_of_Rheumatology
Sun_2006	Therapeutic effect and impact on cytokine production by methotrexate in rheumatoid arthritis. [Chinese]	Beijing_da_xue_xue_bao
Sun_2014	A combination of Sinomenine and Methotrexate reduces joint damage of collagen induced arthritis in rats by modulating osteoclast-related cytokines	International_Immunopharmacology
Suponitskaia_2004	Effect of small-dose glucocorticoids on the course of early rheumatic arthritis. [Russian]	Klinicheskaia_meditsina
Suszko_2013	Influence of polysaccharide fractions isolated from Caltha palustris L. on the cellular immune response in collagen-induced arthritis (CIA) in mice. A comparison with methotrexate	Journal_of_Ethnopharmacology
Suzuki_1997	Short term low dose methotrexate ameliorates abnormal bone metabolism and bone loss in adjuvant induced arthritis	Journal_of_Rheumatology
Svensson_2003	Reumacon (CPH82) showed similar x-ray progression and clinical effects as methotrexate in a two year comparative study on patients with early rheumatoid arthritis	Scandinavian_Journal_of_Rheumatology
Swierkot_2000	Efficacy and toxicity of low dose methotrexate in rheumatoid arthritis patients: A five years analysis. [Polish]	Polskie_Archiwum_Medycyny_Wewnetrznej
Szanto_1986	Low-dose methotrexate in rheumatoid arthritis: Effect and tolerance. An open trial and a double-blind randomized study	Scandinavian_Journal_of_Rheumatology
Szanto_1989	Low-dose methotrexate treatment of rheumatoid arthritis; long-term observation of efficacy and safety	Clin_Rheumatol
Tait_1994	A clinical and biochemical assessment of methotrexate in rheumatoid arthritis	Clinical_Rheumatology
Tak_2011	Inhibition of joint damage and improved clinical outcomes with rituximab plus methotrexate in early active rheumatoid arthritis: The IMAGE trial	Ann_Rheum_Dis
Tak_2012	Sustained inhibition of progressive joint damage with rituximab plus methotrexate in early active rheumatoid arthritis: 2-Year results from the randomised controlled trial IMAGE	Ann_Rheum_Dis
Takeuchi_2013a	A phase 3 randomized, double-blind, multicenter comparative study evaluating the effect of etanercept versus methotrexate on radiographic outcomes, disease activity, and safety in Japanese subjects with active rheumatoid arthritis	Modern_Rheumatology
Takeuchi_2013b	Phase II dose-response study of abatacept in Japanese patients with active rheumatoid arthritis with an inadequate response to methotrexate	Modern_Rheumatology

Takeuchi_2014	Adalimumab, a human anti-TNF monoclonal antibody, outcome study for the prevention of joint damage in Japanese patients with early rheumatoid arthritis: The HOPEFUL 1 study	Ann_Rheum_Dis
Talaat_2015	Therapeutic effect of dimethyl dimethoxy biphenyl dicarboxylate on collagen-induced arthritis in rats	Chinese_Journal_of_Integrative_Medicine
Tam_2012	Infliximab is associated with improvement in arterial stiffness in patients with early rheumatoid arthritis - A randomized trial	Journal_of_Rheumatology
Tanaka_2012	Golimumab in combination with methotrexate in Japanese patients with active rheumatoid arthritis: Results of the GO-FORTH study	Ann_Rheum_Dis
Tanaka_2015a	Clinical efficacy, radiographic progression, and safety through 156 weeks of therapy with subcutaneous golimumab in combination with methotrexate in Japanese patients with active rheumatoid arthritis despite prior methotrexate therapy: final results of the randomized GO-FORTH trial	Mod_Rheumatol
Tanaka_2015b	Prevention of joint destruction in patients with high disease activity or high C-reactive protein s: Post hoc analysis of the GO-FORTH study	Mod_Rheumatol
Tanaka_2016	Efficacy and Safety of Baricitinib in Japanese Patients with Active Rheumatoid Arthritis Receiving Background Methotrexate Therapy: A 12-week, Double-blind, Randomized Placebo-controlled Study	J_Rheumatol
Tascioglu_2003	The effect of low-dose methotrexate on bone mineral density in patients with early rheumatoid arthritis	Rheumatology_International
Taylor_2004	Comparison of Ultrasonographic Assessment of Synovitis and Joint Vascularity With Radiographic Evaluation in a Randomized, Placebo-Controlled Study of Infliximab Therapy in Early Rheumatoid Arthritis	Arthritis_Rheumatism
Taylor_2006	Ultrasonographic and radiographic results from a two-year controlled trial of immediate or one-year-delayed addition of infliximab to ongoing methotrexate therapy in patients with erosive early rheumatoid arthritis	Arthritis_Rheumatism
Taylor_2011a	Maintenance of efficacy and safety with subcutaneous golimumab among patients with active rheumatoid arthritis who previously received intravenous golimumab	Journal_of_Rheumatology
Taylor_2011b	Ofatumumab, a fully human anti-CD20 monoclonal antibody, in biological-naive, rheumatoid arthritis patients with an inadequate response to methotrexate: A randomised, double-blind, placebo-controlled clinical trial	Ann_Rheum_Dis
Tchetina_2013	Rheumatoid factor positivity is associated with increased joint destruction and upregulation of matrix metalloproteinase 9 and cathepsin K gene expression in the peripheral blood in rheumatoid arthritic patients treated with methotrexate	International_Journal_of_Rheumatology
Tchetverikov_2008	Leflunomide and methotrexate reduce s of activated matrix metalloproteinases in complexes with alpha<inf>2</inf> macroglobulin in serum of rheumatoid arthritis patients	Ann_Rheum_Dis
Tekeoglu_2007	Effects of thymoquinone (volatile oil of black cumin) on rheumatoid arthritis in rat models	Phytotherapy_Research

Teramachi_2011	Adenosine abolishes MTX-induced suppression of osteoclastogenesis and inflammatory bone destruction in adjuvant-induced arthritis	Laboratory_Investigation
Thite_2014	Anti-arthritic activity profile of methanolic extract of Ficus bengalensis: Comparison with some clinically effective drugs	Biomedicine_Aging_Pathology
Thomas_1993	Reduction of leukocyte and interleukin-1 beta concentrations in the synovial fluid of rheumatoid arthritis patients treated with methotrexate	Arthritis_Rheumatism
To_2011	Methotrexate chronotherapy is effective against rheumatoid arthritis	Chronobiology_International
Torikai_2006	The effect of methotrexate on bone metabolism markers in patients with rheumatoid arthritis	Mod_Rheumatol
Torrance_2004	Improvement in health utility among patients with rheumatoid arthritis treated with adalimumab (a human anti-TNF monoclonal antibody) plus methotrexate	Rheumatology
ToS_2009	Therapeutic index of methotrexate depends on circadian cycling of tumour necrosis factor-alpha in collagen-induced arthritic rats and mice	Journal_of_Pharmacy_Pharmacology
Trampisch_2014	Comparison of the efficacy and safety of two starting dosages of prednisolone in early active rheumatoid arthritis (CORRA): study protocol for a randomized controlled trial	Trials
Tugwell_1990	Methotrexate in rheumatoid arthritis. Impact on quality of life assessed by traditional standard-item and individualized patient preference health status questionnaires	Archives_of_Internal_Medicine
Tugwell_1995	Combination therapy with cyclosporine and methotrexate in severe rheumatoid arthritis	New_England_Journal_of_Medicine
Tugwell_2000	Clinical improvement as reflected in measures of function and health- related quality of life following treatment with leflunomide compared with methotrexate in patients with rheumatoid arthritis: Sensitivity and relative efficiency to detect a treatment effect in a twelve-month, placebo-controlled trial	Arthritis_Rheumatism
Urata_2007	Effect of a disease-modifying antirheumatic drug iguratimod (T-614) on chronic arthritis in experimental animals. [Japanese]	Japanese_Pharmacology_Therapeutics
Usova_1993	[A comparative evaluation of the treatment results with cyclosporin A, methotrexate and azathioprine in rheumatoid arthritis patients (a preliminary report)]	Ter_Arkh
VanAken_2014	Five-year outcomes of probable rheumatoid arthritis treated with methotrexate or placebo during the first year (the PROMPT study)	Ann_Rheum_Dis
VanDerHeijde_2005a	Patient reported outcomes in a trial of combination therapy with etanercept and methotrexate for rheumatoid arthritis: The TEMPO trial	Ann_Rheum_Dis
VanDerHeijde_2005b	Presentation and analysis of data on radiographic outcome in clinical trials: Experience from the TEMPO study	Arthritis_Rheumatism
VanDerHeijde_2005c	Comparison of different definitions to classify remission and sustained remission: 1 Year TEMPO results	Ann_Rheum_Dis
VanDerHeijde_2006	Comparison of etanercept and methotrexate, alone and combined, in the treatment of rheumatoid arthritis: Two-year clinical and radiographic results from the TEMPO study, a double-blind, randomized trial	Arthritis_Rheumatism
VanDerHeijde_2007	Disease remission and sustained halting of radiographic progression with combination etanercept and methotrexate in patients with rheumatoid arthritis	Arthritis_Rheumatism

VanDerHeijde_2010	Disease activity, physical function, and radiographic progression after longterm therapy with adalimumab plus methotrexate: 5-Year results of PREMIER	Journal_of_Rheumatology
VanDerHeijde_2013	Tofacitinib (CP-690,550) in patients with rheumatoid arthritis receiving methotrexate: Twelve-month data from a twenty-four-month phase III randomized radiographic study	Arthritis_Rheumatism
VanDerKooij_2009	Patient-reported outcomes in a randomized trial comparing four different treatment strategies in recent-onset rheumatoid arthritis	Arthritis_Care_Research
VanDerVeen_1993	The effect of methylprednisolone pulse therapy on methotrexate treatment of rheumatoid arthritis	Clinical_Rheumatology
VanDongen_2007	Efficacy of methotrexate treatment in patients with probable rheumatoid arthritis: A double-blind, randomized, placebo-controlled trial	Arthritis_Rheumatism
VanEde_2001	Effect of folic or folinic acid supplementation on the toxicity and efficacy of methotrexate in rheumatoid arthritis: A forty-eight-week, multicenter, randomized, double-blind, placebo-controlled study	Arthritis_Rheumatism
VanEde_2002a	Homocysteine and folate status in methotrexate-treated patients with rheumatoid arthritis	Rheumatology
VanEde_2002b	The effect of folic- or folinic-acid comedication on the toxicity and efficacy of methotrexate in the treatment of patients with rheumatoid arthritis; a 48-week, randomised, double-blind, placebo-controlled study. [Dutch]	Nederlands_Tijdschrift_voor_Geneeskunde
VanEde_2002c	Purine enzymes in patients with rheumatoid arthritis treated with methotrexate	Ann_Rheum_Dis
VanGestel_1998	Validation of rheumatoid arthritis improvement criteria that include simplified joint s	Arthritis_Rheumatism
VanHolten_2005	A multicentre, randomised, double blind, placebo controlled phase II study of subcutaneous interferon beta-1a in the treatment of patients with active rheumatoid arthritis	Ann_Rheum_Dis
VanRiel_2003	Leflunomide improves the clinical response in patients with active rheumatoid arthritis treated with methotrexate	Clinical_experimental_rheumatology
VanVollenhoven_2010	Improvement in work place and household productivity for patients with early rheumatoid arthritis treated with adalimumab plus methotrexate: work outcomes and their correlations with clinical and radiographic measures from a randomized controlled trial companion study	Arthritis_Care_Research
VanVollenhoven_2011	American college of rheumatology hybrid analysis of certolizumab pegol plus methotrexate in patients with active rheumatoid arthritis: Data from a 52-week phase iii trial	Arthritis_Care_Research
vanVollenhoven_2012	Tofacitinib or adalimumab versus placebo in rheumatoid arthritis	New_England_Journal_of_Medicine
VanVollenhoven_2015	Brief report: Enhancement of patient recruitment in rheumatoid arthritis clinical trials using a multi-biomarker disease activity score as an inclusion criterion	Arthritis_Rheumatology
VanVollenhoven_2016	Full dose, reduced dose or discontinuation of etanercept in rheumatoid arthritis	Ann_Rheum_Dis
Vergunst_2009	MLN3897 plus methotrexate in patients with rheumatoid arthritis: Safety, efficacy, pharmacokinetics, and pharmacodynamics of an oral CCR1 antagonist in a phase IIa, double-blind, placebo-controlled, randomized, proof-of-concept study	Arthritis_Rheumatism

Verschueren_2015	Patients lacking classical poor prognostic markers might also benefit from a step-down glucocorticoid bridging scheme in early rheumatoid arthritis: Week 16 results from the randomized multicenter CareRA trial	Arthritis_Res_Ther
Verstappen_2005	A good response to early DMARD treatment of patients with rheumatoid arthritis in the first year predicts remission during follow up	Ann_Rheum_Dis
Verstappen_2007	Intensive treatment with methotrexate in early rheumatoid arthritis: Aiming for remission. Computer Assisted Management in Early Rheumatoid Arthritis (CAMERA, an open-label strategy trial)	Ann_Rheum_Dis
Vincenzi_2014	Adenosine Receptors Are Differentially Modulated by Pharmacological Treatments in Rheumatoid Arthritis Patients and Their Stimulation Ameliorates Adjuvant-Induced Arthritis in Rats	PLoS_ONE
Visvanathan_2006	IgG and IgM anticardiolipin antibodies following treatment with infliximab plus methotrexate in patients with early rheumatoid arthritis	Arthritis_Rheumatism
Visvanathan_2007	Changes in biomarkers of inflammation and bone turnover and associations with clinical efficacy following infliximab plus methotrexate therapy in patients with early rheumatoid arthritis	Journal_of_Rheumatology
Visvanathan_2009	E-selectin, interleukin 18, serum amyloid A, and matrix metalloproteinase 9 are associated with clinical response to golimumab plus methotrexate in patients with active rheumatoid arthritis despite methotrexate therapy	Journal_of_Rheumatology
Wada_2005	R-130823, a novel inhibitor of p38 MAPK, ameliorates hyperalgesia and swelling in arthritis models	European_Journal_of_Pharmacology
Wagner_2013	Evaluation of serum biomarkers associated with radiographic progression in methotrexate-naive rheumatoid arthritis patients treated with methotrexate or golimumab	Journal_of_Rheumatology
Wahba_2015	Protective effects of fenofibrate and resveratrol in an aggressive model of rheumatoid arthritis in rats	Pharm_Biol
Wan_2013	Effect of tripterygium glycosides on pulmonary function in adjuvant arthritisrats	Journal_of_the_Chinese_Medical_Association
Wang_1994	Effect of total glucoside of paeony on rheumatoid arthritis patients. [Chinese]	Chinese_Pharmacological_Bulletin
Wang_2004	Effect of bizhongxiao decotion on TNF-alpha and interleukin-1beta in plasma of rats with C II-induced rheumatoid arthritis. [Chinese]	Zhong_nan_da_xue_xue_bao
Wang_2005	Effect of Bizhongxiao Decotion (BZXD) on Some Cytokines in Plasma of Rats with CII-induced Rheumatoid Arthritis	Int_J_Biomed_Sci
Wang_2007	Effect of the external use of flexible nanonipsomes methotrexate on the s of serum interferon-gamma and interleukin-4 in rats with rheumatoid arthritis. [Chinese]	J_Clin_Rehab_Tissue_Eng_Res
Wang_2012	(1)H NMR-based metabolomic analysis for identifying serum biomarkers to evaluate methotrexate treatment in patients with early rheumatoid arthritis	Exp_Ther_Med
Wang_2013	Angiotensin II type 2 receptor correlates with therapeutic effects of losartan in rats with adjuvant-induced arthritis	Journal_of_Cellular_Molecular_Medicine
Wei_2009	A multicenter, double-blind, randomized, controlled phase III clinical trial of chicken type II collagen in rheumatoid arthritis	Arthritis_Res_Ther
Weinblatt_1985	Efficacy of low-dose methotrexate in rheumatoid arthritis	New_England_Journal_of_Medicine
Weinblatt_1991	Methotrexate in rheumatoid arthritis: effects on disease activity in a multicenter prospective study	J_Rheumatol

Weinblatt_1992	Long-term prospective study of methotrexate in the treatment of rheumatoid arthritis. 84-month update	Arthritis_Rheumatism
Weinblatt_1993	The effects of drug therapy on radiographic progression of rheumatoid arthritis. Results of a 36-week randomized trial comparing methotrexate and auranofin	Arthritis_Rheumatism
Weinblatt_1994	Methotrexate in rheumatoid arthritis: A five-year prospective multicenter study	Arthritis_Rheumatism
Weinblatt_1998	Longterm prospective study of methotrexate in rheumatoid arthritis: Conclusion after 132 months of therapy	Journal_of_Rheumatology
Weinblatt_1999	A trial of etanercept, a recombinant tumor necrosis factor receptor:Fc fusion protein, in patients with rheumatoid arthritis receiving methotrexate	New_England_Journal_of_Medicine
Weinblatt_2003	Adalimumab, a fully human anti-tumor necrosis factor alpha monoclonal antibody, for the treatment of rheumatoid arthritis in patients taking concomitant methotrexate: The ARMADA trial	Arthritis_Rheumatism
Weinblatt_2006	Long term efficacy and safety of adalimumab plus methotrexate in patients with rheumatoid arthritis: ARMADA 4 year extended study	Ann_Rheum_Dis
Weinblatt_2011	Factors associated with radiographic progression in patients with rheumatoid arthritis who were treated with methotrexate	Journal_of_Rheumatology
Weinblatt_2013a	Effects of fostamatinib (R788), an oral spleen tyrosine kinase inhibitor, on health-related quality of life in patients with active rheumatoid arthritis: Analyses of patient-reported outcomes from a randomized, double-blind, placebo-controlled trial	Journal_of_Rheumatology
Weinblatt_2013b	Intravenous golimumab is effective in patients with active rheumatoid arthritis despite methotrexate therapy with responses as early as week 2: Results of the phase 3, randomised, multicentre, double-blind, placebo-controlled GO-FURTHER trial	Ann_Rheum_Dis
Weinblatt_2013c	Radiographic benefit and maintenance of clinical benefit with intravenous golimumab therapy in patients with active rheumatoid arthritis despite methotrexate therapy: results up to 1 year of the phase 3, randomised, multicentre, double blind, placebo controlled GO-FURTHER trial	Ann_Rheum_Dis
Weinblatt_2015	The efficacy and safety of subcutaneous clazakizumab in patients with moderate-to-severe rheumatoid arthritis and an inadequate response to methotrexate: results from a multinational, phase IIb, randomized, double-blind, placebo/active-controlled, dose-ranging study	Arthritis_Rheumatol
Weisman_2003	Efficacy, pharmacokinetic, and safety assessment of adalimumab, a fully human anti-tumor necrosis factor-alpha monoclonal antibody, in adults with rheumatoid arthritis receiving concomitant methotrexate: A pilot study	Clinical_Therapeutics
Welles_1985	Studies on the effect of low dose methotrexate on rat adjuvant arthritis	Journal_of_Rheumatology
Wells_2011	Abatacept plus methotrexate provides incremental clinical benefits versus methotrexate alone in methotrexate-naive patients with early rheumatoid arthritis who achieve radiographic nonprogression	Journal_of_Rheumatology
Wessels_2006	Efficacy and toxicity of methotrexate in early rheumatoid arthritis are associated with single-nucleotide polymorphisms in genes coding for folate pathway enzymes	Arthritis_Rheumatism
Wessels_2007	A clinical pharmacogenetic model to predict the efficacy of methotrexate monotherapy in recent-onset rheumatoid arthritis	Arthritis_Rheumatism

Westedt_1994a	Azathioprine compared with methotrexate for rheumatoid arthritis: An open randomized clinical trial	Revue_du_Rhumatisme_(English_Edition)
Westedt_1994b	Comparison of azathioprine and methotrexate in rheumatoid arthritis: an open-randomized clinical study. [French]	Revue_du_rhumatisme_(Ed
Westhovens_2006	The safety of infliximab, combined with background treatments, among patients with rheumatoid arthritis and various comorbidities: A large, randomized, placebo-controlled trial	Arthritis_Rheumatism
Westhovens_2009	Clinical efficacy and safety of abatacept in methotrexate-naive patients with early rheumatoid arthritis and poor prognostic factors	Ann_Rheum_Dis
Westhovens_2013	Oral administration of GLPG0259, an inhibitor of MAPKAPK5, a new target for the treatment of rheumatoid arthritis: a phase II, randomised, double-blind, placebo-controlled, multicentre trial	Ann_Rheum_Dis
Westhovens_2014	Long-term safety and efficacy of abatacept in patients with rheumatoid arthritis and an inadequate response to methotrexate: A 7-year extended study	Clinical_Experimental_Rheumatology
Wijngaarden_2005	Down-regulation of activating Fcgamma receptors on monocytes of patients with rheumatoid arthritis upon methotrexate treatment	Rheumatology
Williams_1985	Comparison of low-dose oral pulse methotrexate and placebo in the treatment of rheumatoid arthritis. A controlled clinical trial	Arthritis_Rheumatism
Williams_1992	Comparison of auranofin, methotrexate, and the combination of both in the treatment of rheumatoid arthritis. A controlled clinical trial	Arthritis_Rheumatism
Willkens_1982	Methotrexate: a perspective of its use in the treatment of rheumatic diseases	J_Lab_Clin_Med
Willkens_1992	Comparison of azathioprine, methotrexate, and the combination of both in the treatment of rheumatoid arthritis. A controlled clinical trial	Arthritis_Rheumatism
Willkens_1995	Comparison of azathioprine, methotrexate, and the combination of the two in the treatment of rheumatoid arthritis: A forty-eight-week controlled clinical trial with radiologic outcome assessment	Arthritis_Rheumatism
Willkens_1996	Combination treatment of rheumatoid arthritis using azathioprine and methotrexate: A 48 week controlled clinical trial	Journal_of_Rheumatology
Wislowska_2007	Preliminary evaluation in rheumatoid arthritis activity in patients treated with TNF-alpha blocker plus methotrexate versus methotrexate or leflunomide alone	Rheumatol_Int
Wisniacki_2013	Safety, tolerability, pharmacokinetics, and pharmacodynamics of anti-TWEAK monoclonal antibody in patients with rheumatoid arthritis	Clinical_Therapeutics
Wunder_2004	In vivo imaging of protease activity in arthritis: A novel approach for monitoring treatment response	Arthritis_Rheumatism
Xi_2010	Effect of thalidomide on inflammatory factor expression in rats with collagen induced arthritis. [Chinese]	Journal_of_Clinical_Rehabilitative_Tissue_Engineering_Resear ch
Xia_2011	Blockage of TNF-alpha by infliximab reduces CCL2 and CCR2 s in patients with rheumatoid arthritis	J_Investig_Med
Xia_2015	Iguratimod in combination with methotrexate in active rheumatoid arthritis : Therapeutic effects	Z_Rheumatol
Xiao_2010	Associations between the genetic polymorphisms of MTHFR and outcomes of methotrexate treatment in rheumatoid arthritis	Clin_Exp_Rheumatol

Xinqiang_2010	Therapeutic efficacy of experimental rheumatoid arthritis with low-dose methotrexate by increasing partially CD4⁺CD25⁺Treg cells and inducing Th1 to Th2 shift in both cells and cytokines	Biomedicine_Pharmacotherapy
Xu_2011	Anti-angiogenic effects of genistein on synovium in a rat model of type II collagen-induced arthritis. [Chinese]	Journal_of_Chinese_Integrative_Medicine
Xu_2015	Therapeutic effects of micheliolide on a murine model of rheumatoid arthritis	Molecular_Medicine_Reports
Yamaki_2003	Effect of methotrexate on Th1 and Th2 immune responses in mice	Journal_of_Pharmacy_Pharmacology
Yamaki_2005	Effect of varying types of anti-arthritic drugs on TH1 and TH2 immune responses in mice	International_Journal_of_Immunopathology_Pharmacology
Yamamoto_2014	Efficacy and safety of certolizumab pegol plus methotrexate in Japanese rheumatoid arthritis patients with an inadequate response to methotrexate: the J-RAPID randomized, placebo-controlled trial	Modern_Rheumatology
Yamanaka_2015	Discontinuation of etanercept after achievement of sustained remission in patients with rheumatoid arthritis who initially had moderate disease activity-results from the ENCOURAGE study, a prospective, international, multicenter randomized study	Mod_Rheumatol
Yao_2013	Combination of MTX and LEF attenuates inflammatory bone erosion by down-regulation of receptor activator of NF-kB ligand and interleukin-17 in type II collagen-induced arthritis rats	Rheumatology_International
Yellin_2012	A phase II, randomized, double-blind, placebo-controlled study evaluating the efficacy and safety of MDX-1100, a fully human anti-CXCL10 monoclonal antibody, in combination with methotrexate in patients with rheumatoid arthritis	Arthritis_Rheumatism
Yount_2007	Adalimumab plus methotrexate or standard therapy is more effective than methotrexate or standard therapies alone in the treatment of fatigue in patients with active, inadequately treated rheumatoid arthritis	Clinical_Experimental_Rheumatology
Yu_2013	Combination with methotrexate and cyclophosphamide attenuated maturation of dendritic cells: Inducing treg skewing and Th17 suppression in vivo	Clinical_Developmental_Immunology
Yue_2010	The effects of adalimumab and methotrexate treatment on peripheral Th17 cells and IL-17/IL-6 secretion in rheumatoid arthritis patients	Rheumatol_Int
Zhang_2006	Infliximab versus placebo in rheumatoid arthritis patients receiving concomitant methotrexate: A preliminary study from China	APLAR_Journal_of_Rheumatology
Zhang_2008	A randomized, double-blind, multicenter, controlled clinical trial of chicken type II collagen in patients with rheumatoid arthritis	Arthritis_Care_Research
Zhang_2010	A proof-of-concept and drug-drug interaction study of pamapimod, a novel p38 MAP kinase inhibitor, with methotrexate in patients with rheumatoid arthritis	Journal_of_clinical_pharmacology
Zhang_2013a	Comparative efficacy of TACI-Ig with TNF-alpha inhibitor and methotrexate in DBA/1 mice with collagen-induced arthritis	European_Journal_of_Pharmacology
Zhang_2013b	[Efficacy and safety evaluation of fire needling for rats with rheumatoid arthritis]. [Chinese]	Zhongguo_zhen_jiu_=_Chinese_acupuncture_&_moxibustion

Zhao_2009	Therapeutic effects of different doses of recombinant human tumor necrosis factor-receptor II: IgG Fc fusion protein on rheumatoid arthritis. [Chinese]	Journal_of_Shanghai_Jiaotong_University_(Medical_Science)
Zhao_2012	[Short-term clinical observation on compound Xiatianwu combined with methotrexate in treating rheumatoid arthritis]. [Chinese]	Zhongguo_Zhong_Xi_Yi_Jie_He_Za_Zhi
Zhao_2015	Therapeutic effects of water extract of Arisaema erubescens tubers on type II collagen-induced arthritis in rats	Tropical_Journal_of_Pharmaceutical_Research
Zheng_2015	The effect of curcumin and its nanoformulation on adjuvant-induced arthritis in rats	Drug_Design,_Development_Therapy
Zhou_2010a	Synergistic effect of antiangiogenic nanotherapy combined with methotrexate in the treatment of experimental inflammatory arthritis	Nanomedicine
Zhou_2010b	[Clinical study on active rheumatoid arthritis treated with simiao xiaobi decoction]. [Chinese]	Zhongguo_Zhong_Xi_Yi_Jie_He_Za_Zhi
Zhu_2012	Overexpression of Toll-Like Receptor 3 in Spleen is Associated with Experimental Arthritis in Rats	Scandinavian_Journal_of_Immunology
Zuo_2009	[Clinical efficacy of Corydalis composite combined with methotrexate in treating rheumatoid arthritis]. [Chinese]	Zhongguo_Zhong_Xi_Yi_Jie_He_Za_Zhi
Zykova_2007	Antirheumatoid activity of methotrexate in phospholipid nanoparticles (phosphogliv). [Russian]	Biomeditsinskaya_Khimiya
Zykova_2008	Antirheumatic activity of methotrexate in phospholipid nanoparticles (Phosphogliv)	BiochemistrySupplement_Series_B:_Biomedical_Chemistry
